# Slow-growing cells within isogenic populations have increased RNA polymerase error rates and DNA damage

**DOI:** 10.1038/ncomms8972

**Published:** 2015-08-13

**Authors:** David van Dijk, Riddhiman Dhar, Alsu M. Missarova, Lorena Espinar, William R. Blevins, Ben Lehner, Lucas B. Carey

**Affiliations:** 1 Department of Biological Sciences, Columbia University, New York, New York 10027, USA; 2 Department of Systems Biology, Columbia University, New York, New York 10027, USA; 3 Department of Applied Mathematics, Weizmann Institute of Science, Rehovot, 7610001, Israel; 4 Department of Molecular Cell Biology, Weizmann Institute of Science, Rehovot, 7610001, Israel; 5 EMBL-CRG Systems Biology Research Unit, Centre for Genomic Regulation (CRG), Dr Aiguader 88, 08003 Barcelona, Spain; 6 Universitat Pompeu Fabra, Dr Aiguader 88, 08003 Barcelona, Spain; 7 Department of Experimental and Health Sciences, Universitat Pompeu Fabra, Dr Aiguader 88, 08003 Barcelona Spain; 8 Research Programme on Biomedical Informatics (GRIB), Hospital del Mar Research Institute (IMIM), Universitat Pompeu Fabra, Dr Aiguader 88, 08003 Barcelona, Spain; 9 Institució Catalana de Recerca i Estudis Avançats (ICREA), Pg. Lluis Companys 23, 08010 Barcelona, Spain

## Abstract

Isogenic cells show a large degree of variability in growth rate, even when cultured in the same environment. Such cell-to-cell variability in growth can alter sensitivity to antibiotics, chemotherapy and environmental stress. To characterize transcriptional differences associated with this variability, we have developed a method—FitFlow—that enables the sorting of subpopulations by growth rate. The slow-growing subpopulation shows a transcriptional stress response, but, more surprisingly, these cells have reduced RNA polymerase fidelity and exhibit a DNA damage response. As DNA damage is often caused by oxidative stress, we test the addition of an antioxidant, and find that it reduces the size of the slow-growing population. More generally, we find a significantly altered transcriptome in the slow-growing subpopulation that only partially resembles that of cells growing slowly due to environmental and culture conditions. Slow-growing cells upregulate transposons and express more chromosomal, viral and plasmid-borne transcripts, and thus explore a larger genotypic—and so phenotypic — space.

## Abstract (web summary)

Isogenic cells growing in the same environment show a large degree of variability. Here, by sorting yeast cells based on growth rate, the authors show that the slow-growing subpopulation exhibits stress responses, a high level of transcriptional diversity, and decreased RNA polymerase fidelity.

## Graphical abstract

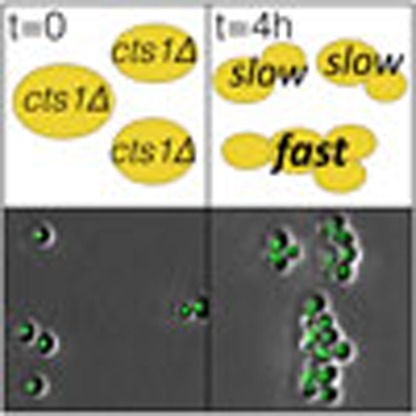

Fitness, in single-cell organisms and cancer, is the number of viable offspring a cell is able to produce in a given amount of time, and is typically measured as a population average trait^1^. However, growth is highly variable (Supplementary Fig. 1)^2^ and any single cell will differ from the population average, resulting in subpopulations that, at least temporarily, maintain a lower growth rate. The presence of such a slow-growing subpopulation has been observed in microbes, metazoans and tumour cells, and has been implicated in persistence, stress sensitivity, bacterial antibiotic resistance^3^,^4^,^5^ and chemoresistance in cancer^6^,^7^,^8^. While changes in growth and its association with changes in gene expression patterns has been extensively studied at the population average level, much less is known about the transcriptional programs of the slow-growing subpopulations.

At the population level, growth rate can be changed environmentally by changing growth condition^9^ or as a result of genetic perturbations^10^,^11^. These changes in growth rate are accompanied by intracellular changes in gene expression. Slow growth is generally associated with a transcriptionally stressed phenotype, whereas fast growth is associated with upregulation of ribosomal genes^9^. Altered mean population-level growth rate has consequences on fitness. Fast-growing *Escherichia coli* are more sensitive to stress and can utilize fewer nutrient sources than their slow-growing counterparts, and this stress sensitivity is correlated to expression of sigma factor RpoS^12^,^13^.

Gene expression shows a large degree of non-genetic within-population variability (noise)^14^,^15^ and as such one would expect this variability to be associated with downstream phenotypes, such as growth. Previous microscopy-based studies have shown that slow- and fast-growing subpopulations differ in the expression level of a few genes^2^,^16^ and that genetic perturbation can change the shape of the growth rate distribution^16^,^17^. However, the general gene expression programs of the slow-growing subpopulation are not at all characterized. This is because existing microscopy-based methods can measure single-cell growth and gene expression for at most three genes at a time, making characterization of large-scale gene expression programs in slow and fast subpopulations a laborious process. In yeast, only a single gene, *TSL1*, is known to correlate with growth rate within a population^16^. Here we present a novel method that enables an unbiased characterization of the transcriptomes of slow and fast subpopulations using fluorescence-activated cell sorting and RNA sequencing (RNA-seq).

Here we describe the development of FitFlow (Fitness Flow cytometry), the first method able to completely characterize subpopulations that differ in their fitness (Fig. 1). We use FitFlow to characterize the transcriptome of slow- and fast-growing subpopulations of budding yeast, and validate the biological conclusions using time-lapse microscopy. We find that the slow-growing subpopulation has a high level of transcriptional diversity: more annotated genes, novel genes and antisense transcripts are transcribed, and genes on the 2-micron plasmid and two endogenous viruses are highly expressed. In addition, RNA polymerase in slow-growing yeast and nematodes is more error prone, resulting in increased messenger RNA (mRNA) diversity at the nucleotide level. Furthermore, the slow-growing subpopulation has higher expression of transposons and shows both a transcriptional and post-translational DNA damage response, suggesting that these cells may have higher rates of transposition and DNA mutation. Addition of vitamin C, an antioxidant, reduced the fraction of slow-growing cells, suggesting that oxidative stress is causative for slow growth in a subset of the slow-growing cells. Taken together, these results suggest that, because the growth rate within a population is highly non-uniform, the potential for epigenetic and genetic changes may be different among cells within a population, in a manner that depends on the growth rate of individual cells.

## Results

### A method to sort yeast by single-cell fitness

**Figure 1:**
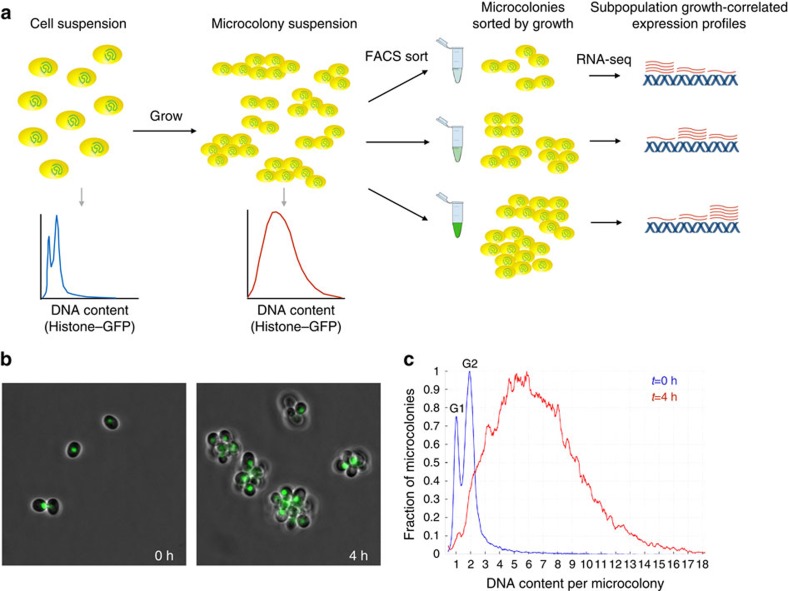
FitFlow: sorting cells by single-cell fitness. (**a**) FitFlow method: single, chitinase-deficient, Hta2–GFP-expressing cells are suspended after brief sonication and G1 selection. Growth for several generations in liquid media results in microcolonies of cells that have a distribution of cell number. Flow cytometry of Hta2–GFP measurement reveals the microcolony size distribution. Subsequent sorting on Hta2–GFP abundance thus separates populations according to their single-cell growth rate. RNA-seq analysis of each bin of growth rate reveals gene expression patterns associated with variable stochastic growth. (**b**) Microscopy at different time points shows microcolony formation. (**c**) Flow cytometry of single cells (*t*=0 h) and microcolonies (*t*=4 h) shows the distribution cell number per microcolony. The HTA2–GFP fusion enables high-resolution measurement of DNA content as shown by separate G1 and G2 peaks at *t*=0 h.

To completely characterize the differences in intracellular state between fast and slow subpopulations in an unbiased manner, we developed a novel method (Fitness flow cytometry, FitFlow) for sorting cells based on quantitative differences in single-cell fitness (the number of progeny a single cell can produce in a given amount of time). In budding yeast, the daughter cell remains attached to the mother cell following cytokinesis with the daughter cell then expressing an enzyme, chitinase, that degrades this linkage^18^ (see Methods). A chitinase knockout (*cts1*Δ) results in cells that do not separate and thus form attached microcolonies of cells descended from a common ancestor (Fig. 1; Supplementary Fig. 2). *cts1*Δ microcolonies are easily separated by brief sonication (Supplementary Fig. 2), enabling us to grow cells in liquid media, break apart the microcolonies and allow them to reform as cells grow and divide. The single-cell fitness distribution can be determined by measuring the distribution of the number of cells per microcolony, using a histone–green fluorescent protein (GFP) fusion protein (HTA2–GFP) (Fig. 1). This method gives results comparable to growth rates measured by time-lapse microscopy (Supplementary Figs 1 and 2). However, in contrast to microscopy, cells can be sorted by fluorescence-activated cell sorting, enabling large-scale characterization of cells that differ by quantitative differences in fitness. We sorted 50,000 microcolonies into each of three bins of single-cell fitness and performed RNA-seq on each sorted population. We found significant transcriptional differences in the slow and fast subpopulations that recapitulate what is known about these two populations in yeast^16^ (Supplementary Fig. 3). In addition, the slow-growing subpopulation expresses more genes involved in alternative carbon and nitrogen source metabolism, suggesting that these cells might be capable of growing on more heterogeneous environments, similar to slow-growing *E. coli*^12^ (Supplementary Fig. 3). This slow growth is a heritable state (Supplementary Fig. 4), however, the slow-growing subpopulation eventually recovers (Supplementary Fig. 5).

### The slow-growing subpopulation expresses more genes

**Figure 2:**
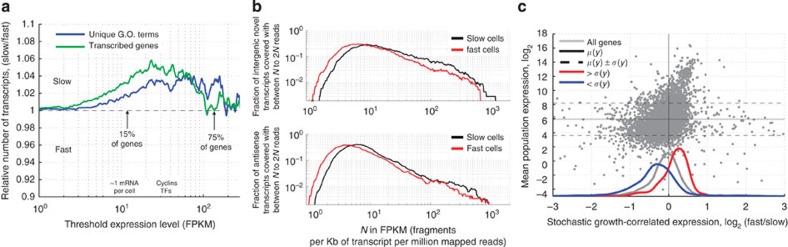
More transcriptional diversity in slow-growing subpopulations. (**a**) At low expression (<5 FPKM (fragments per kilobase of transcript per million mapped reads)), slow- and fast-growing cells express similar numbers of transcripts, but at medium (5–30 FPKM), slow-growing cells express both more genes and more unique gene functions (paired *t*-test *P*<1e−35 for transcripts and GO terms). (**b**) The slow-growing subpopulation expresses more unannotated transcripts (paired ks-test *P*=5.36 × 10^−10^) and antisense transcripts (paired ks-test *P*=1.34 × 10^−22^) at >10 FPKM. (**c**) Highly expressed genes (higher than one s.d., red) are upregulated (paired ks-test *P*=1.1 × 10^−63^), while lowly expressed genes (lower than one s.d., blue) tend to be downregulated with increasing subpopulation growth rate (paired ks-test *P*=4.6 × 10^−35^). *y* axis shows the average expression level in all measured populations. The *x* axis shows expression change from slow to fast subpopulation growth, computed as the log2 ratio.

The faster a population grows, the greater the proportion of the transcriptome is dedicated to ribosome production^9^. In rapidly growing cells, 50% of mRNA synthesis is dedicated to ∼10% of genes^19^. Consistent with these population-level results, we found that, in the fast subpopulation, the most highly expressed genes account for a large fraction of the total transcriptome. In contrast, in the slow subpopulation, the rest of the genome is more highly expressed (Fig. 2). This is not an artefact of detection bias due to fast cells having higher expression of highly expressed genes (Supplementary Figs 6 and 7). In addition to expressing more genes, slow-growing cells also express more unique gene functions (Fig. 2a). An increase in transcription of highly expressed genes results in a far greater commitment of cellular resources than a similar fold change in the transcription of genes with low expression. This suggests that the slow subpopulation shifts resources from high expression of only a few genes to the more moderate expression of a large number of genes. While deletion of most genes does not cause a growth defect in rich media, there exists some condition in which each gene is useful^20^. We find that these slow cells have higher expression of genes that, in fast-growing cells, have little or no expression (Fig. 2). Furthermore, novel (unannotated) and antisense transcripts are expressed at a higher level in the slow-growing subpopulation (Fig. 2b). These results show that the slow-growing subpopulation expresses more genes and unique enzymatic and cellular functions, which may, in turn, allow them to explore a larger phenotypic space.

### Correlation between subpopulation and other transcriptomes

**Figure 3:**
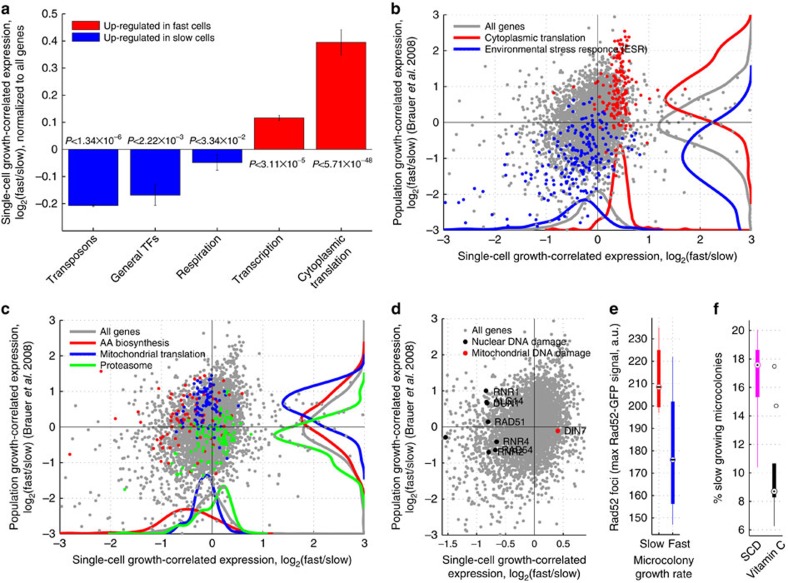
Transcriptional profiles of mean and subpopulation growth. (**a**) Bar-plot showing mean and standard expression of all genes in each functional group of genes upregulated in the slow- (blue) or fast (blue)-growing subpopulations. (**b**–**d**) Growth-correlated expression from slow- and fast-growing subpopulations (FitFlow, *x* axis) are compared with expression differences from growth rate varied in nutrient limited chemostats (*y* axis). (**b**) Scatter-plot the correlation of gene expression between subpopulation growth and mean population growth. Ribosomal genes (red) and stress genes (blue) are, respectively, up- and downregulated both in subpopulation (*x* axis, paired ks-tests *P*_red_=3.36 × 10^−67^; *P*_blue_=4.02 × 10^−21^) and mean population (*y* axis, paired ks-tests *P*_red_=2.45 × 10^−36^; *P*_blue_=8.26 × 10^−50^) fast growth. (**c**) Scatter-plot highlighting genes for which subpopulation growth is anti-correlated with mean population growth. Amino-acid biosynthesis (red) and mitochondrial translation (blue) are downregulated in the fast subpopulation (paired ks-tests *P*_red_=1.03 × 10^−12^; *P*_blue_=3.31 × 10^−04^) but upregulated in mean population fast growth (paired ks-tests *P*_red_=3.30 × 10^−04^; *P*_blue_=2.64 × 10^−16^), while the proteasome (green) is upregulated in the fast subpopulation (paired ks-test *P*=2.25 × 10^−06^) but downregulated in mean population fast growth (paired ks-test *P*=4.69 × 10^−07^). (**d**) DNA damage genes (black points) are upregulated in the slow subpopulation (*x* axis, paired ks-test *P*=4.73 × 10^−07^), but are not correlated with average population growth rate differences (*y* axis, paired ks-test *P*=0.83). DIN7 (red point) is involved in mitochondrial DNA damage repair, and is the only DNA damage related gene that is not upregulated in the slow subpopulation. (**e**) Time-lapse microscopy shows that cells from the slow-growing subpopulation have Rad52–GFP foci. Foci were measured as the maximum Rad52–GFP signal in the nucleus (*y* axis) and growth (*x* axis) as the microcolony growth rate where slow and fast cells represent the slowest 25% and fastest 75%, respectively (*t*-test, *P*=0.02). (**f**) Addition of the antioxidant vitamin C reduced the fraction of slow-growing microcolonies (*t*-test, *P*=0.005).

To better understand the details of this transcriptional shift, we analysed groups of genes that are differentially expressed between slow and fast subpopulations. Genes involved in transcription and cytoplasmic translation are more highly expressed in fast-growing cells; however, the number of expressed transcription factors is actually higher in slow cells (Fig. 3a,b), suggesting that they diversify their transcriptional program by increasing the number of expressed transcription factors. In addition, genes involved in respiration (Fig. 3a) are more highly expressed in slow-growing cells, as are genes involved in mitochondrial translation (Fig. 3c), suggesting that the slow-growing subpopulation is respiring.

Genetic and environmentally determined growth differences show similar changes in transcriptional profiles^9^,^11^. We therefore wondered whether transcriptome changes associated with subpopulation growth differences are similar to differences associated with average population growth rate alteration. For each gene, we computed the log ratio of expression between the fast and slow subpopulation growth bins. We then compared this log ratio (growth associated gene expression change) with the log ratio derived from growth rates altered via nutrient limitation in a chemostat^9^ (see Methods). Both mechanisms of changing growth have a common transcriptional profile: stress genes go up in slow-growing cells, while ribosomal genes go up in fast-growing cells (*r*=0.31, *P*<10^−64^). Similar results are obtained when looking at gene expression as a function of growth rate determined by genetic perturbations^11^ (*r*=0.35, *P*<10^−120^) (Supplementary Fig. 8). To visualize the extent to which subpopulation differences are similar to average population differences, we plot subpopulation growth-correlated gene expression changes (as measured by FitFlow) against average population growth-correlated expression changes (derived from data by Bauer *et al*.) (Fig. 3b–d). In particular, when looking just at the environmental stress response and ribosomal genes, the two transcriptional profiles show high similarity (*r*=0.68, *P*<2e−31). These results suggest that average population and subpopulation growth differences have some similarity at the transcriptional level, but that the slow-growing subpopulations are not identical to environmentally slow-growing cells.

We also observed significant differences between subpopulation and population average growth (Supplementary Data 1); large groups of related genes are differentially regulated in the two data sets (Fig. 3c). While we note that any differences may be due to fundamental differences in the media and growth conditions between batch and chemostat experiments, it is still informative to analyse the differences. While most ribosomal genes are upregulated in the fast-growing subpopulation, the components of the mitochondrial ribosome are more highly expressed in slow cells than in fast cells (Fig. 3c). This is in contrast to population average growth, in which the majority of ribosomal genes, including the ones involved in mitochondrial translation, are upregulated in fast-growing cells (Fig. 3c). In addition, the fast subpopulation downregulates amino-acid biosynthesis genes, whereas in fast average population growth these same genes are relatively upregulated (Fig. 3c). Using data from an experiment in which yeast growing on glucose were shifted to glycerol^21^, we find that amino-acid biosynthesis genes are upregulated when cells are shifted from glucose to glycerol, but not during continuous growth on glycerol (Supplementary Fig. 20), suggesting that the upregulation of these genes may be a response to a rapid decrease in growth rate or part of the switch to respiratory growth. Moreover, proteasomal genes are upregulated in the fast subpopulation but downregulated in fast average population growth (Fig. 3c). To test the differential sensitivity of the subpopulation to proteasome inhibition, we grew *pdr5*Δ cells in the proteasome inhibitor MG132. Faster-growing cells are slightly more sensitive to proteasome inhibition (Supplementary Fig. 9); the growth rate of the fast majority is decreased but the size of the slow tail remains the same.

### Slow growers have oxidative stress and DNA damage

Stress may promote genomic alterations^22^,^23^,^24^,^25^. Consistent with this, we found that transposons are highly expressed in the slow subpopulation (Fig. 3a), but not in the slow average population (Supplementary Fig. 10). In addition, out of the nine genes that are upregulated specifically in response to environmental DNA damage^26^, eight are upregulated in the slow-growing subpopulation (Fig. 3d). The exception, *DIN7*, is involved only in mitochondrial DNA damage repair^27^.

To determine whether slow cells have increased nuclear DNA damage, we measured Rad52–GFP foci^28^ using time-lapse microscopy. We found that cells in the slow-growing subpopulation have Rad52 foci (Fig. 3e), and that cells with a Rad52 focus show a short-term decrease in growth rate (Supplementary Fig. 12). This suggests either that slow growth is causative of an increase in DNA damage or that DNA damage sometimes, but not always, causes cells to grow slowly. Oxidative stress can cause DNA damage^29^. We therefore grew wild-type yeast in the presence of the water-soluble antioxidant L-ascorbic acid (vitamin C). We found that vitamin C decreases the fraction of slow-growing microcolonies (Fig. 3f; Supplementary Fig. 13). These results suggest that DNA damage and/or oxidative stress may be a cause of slow subpopulation growth.

### Stresses alter the single-cell fitness distribution

The above results suggest that internal stresses, such as DNA damage or oxidative stress may result in heritable changes in growth rates. To determine whether extracellular stresses can alter the growth rate distribution, we grew wild-type cells in different environmental stresses and measured the distribution using microscopy. We found that stresses can alter the growth rate distribution independent of their effect on the mode (Supplementary Figs 14–16). For example, 7 mM MnCl and 100 mM LiAc result in the same mode growth rate, but MnCl results in a far wider distribution (Supplementary Fig. 14). Addition of H_2_O_2_ across a 200-fold range resulted in a 20% decrease in growth rate but no change in the shape of the distribution (Supplementary Fig. 16). None of the conditions tested (save vitamin C) results in the decrease in the slow-growing subpopulation, consistent with the transcriptomics data suggesting that these cells are already stressed.

### Slow-growing cells express more selfish DNA elements

**Figure 4:**
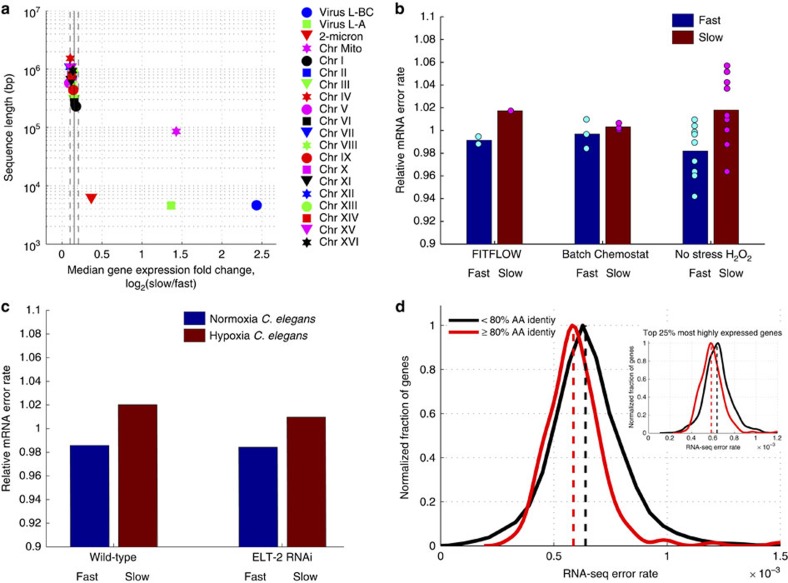
Slow-growing cells exhibit more transcript errors. (**a**) For each genomic nucleic acid molecule (plasmid, chromosome or RNA virus), the median expression fold change, across all genes (*x* axis), is graphed versus nucleic acid sequence length. Vertical solid and dashed lines show mean and s.d. of the 16 native yeast chromosomes. Mitochondria (magenta star), two viruses (blue circle and green square) and the 2-micron plasmid (red triangle) show significantly stronger upregulation in slow cells compared with the 16 yeast chromosomes. (**b**) The number of RNA-seq errors was measured across the genome for three different experiments performed in three different labs. RNA-seq data from isogenic cells that differ only by their stochastic growth rate, the growth conditions, or have been grown in H_2_O_2_ for 30 min, were analysed to measure the amount per-nucleotide transcriptome variability in each condition. In all cases, slow-growing cells have more variability (*t*-test, *P*=0.013 for the H_2_O_2_ data, *t*-test *P*=0.004 for all the data combined. (**c**) To determine whether this effect is yeast specific, an identical genome-wide analysis was performed on RNA-seq data from *C. elegans* subjected to 0.5% O_2_ for 36 h. These hypoxia stressed nematodes also contain more errors in their transcriptomes. (**d**) More highly conserved genes (% amino acid identity across yeast species^53^) have lower RNA-seq error rates (average error rate across all experiments) suggesting a lower RNA polymerase II error rate (*t*-test, *P*<10^−3^). This is not a function of expression level; the correlation with amino-acid conservation is seen when looking at only the top 25% of genes by expression level (inset, *P*<10^−6^).

Additional selfish genetic entities other than transposons can also be induced by stress^30^, and might contribute to further genic diversity. We found that genes on the 2-micron plasmid, a small (∼6 kb) selfish plasmid, and from narnaviruses, a form of selfish RNA are more highly expressed in the slow subpopulation (Fig. 4a). In addition to transposons, the slow-growing subpopulation exhibits higher expression of plasmid-borne and viral genes. The same higher expression of selfish DNA elements was also observed in slow-growing cells in a chemostat (Supplementary Fig. 11).

### Slow-growing cells have lower RNA polymerase fidelity

Errors during gene expression can directly cause phenotypic diversity and, at least in some cases, error-induced phenotypic change can be epigenetically heritable due to the reprogramming of transcriptional networks^31^. To determine whether the slow subpopulation exhibits more transcriptional diversity at the nucleotide level, we calculated the frequency of RNA-seq mismatches to the reference genome. We find that the slow-growing subpopulation has more RNA-seq mismatches with the reference genome, suggesting decreased RNA polymerase fidelity and, therefore, increased transcriptional diversity at the nucleotide level (Fig. 4b). While most mismatches are technical errors, the technical error rate is highly reproducible within an experiment (Supplementary Fig. 17), and can be treated as a constant.

To test whether the observed increased mRNA error rate is a general property of slow-growing and or stressed cells, we analysed additional RNA-seq data sets in which slow- and fast-growing cells were compared: yeast growing with and without H_2_O_2_ (ref. 32), and yeast growing in excess glucose (batch culture) versus glucose limitation (in a chemostat)^33^. In all cases, the slow culture has more sequence errors (Fig. 4b), suggesting that increased mRNA sequence variation is a property of slow-growing cells.

To determine whether this is a microbe-specific phenotype, we analysed data from an experiment in which nematodes were grown in 0.5% oxygen (hypoxia stress)^34^. Stressed *Caenorhabditis elegans* also exhibit an increased RNA polymerase error rate in response to stress (Fig. 4c), suggesting that this may be part of a general stress response across all organisms. This change in error rate is constant across all levels of RNA-seq coverage and is not due to differential gene expression (Supplementary Fig. 17).

The increased transcriptome error rate in slow-growing and stressed cells could stem from either an increased RNA polymerase II error rate or an increase in genomic DNA mutations. We find that more highly conserved genes have a lower transcriptional error rate (Fig. 4d), suggesting that error rate is a regulated process. There are three reasons to favour RNA polymerase as the source of these errors. First, the DNA polymerase error rate^35^ is ∼5e^−10^, while the RNA polymerase II error rate^36^,^37^ is ∼10^−5^ and our measured change is ∼3e^−5^. The increase in errors that we detect in slow-growing cells is similar to the 30% increase in RNA polymerase error rate observed in an *rbp9* strain^36^, but an increase of >100,000-fold in the DNA polymerase error rate. Second, in the H_2_O_2_ stressed cells, the increased error rate is detected within 30 min, which is not consistent with DNA polymerase as a source of errors. Third, *RBP9*, which is involved in RNA polymerase II fidelity *in vivo*^36^, is downregulated in the slow-growing subpopulation more than the rest of the RNA polymerase complex (Supplementary Fig. 17), suggesting imbalanced polymerase II stoichiometry as a possible mechanism for reduced transcriptional fidelity.

## Discussion

In summary, by developing a method to physically sort slow-growing subpopulations, we have presented here the first global and unbiased view of changes in cellular state associated with differences in growth rate within one isogenic population growing in the same environment. Transcriptome analysis suggests that cells from the slow-growing subpopulation are respiring and exhibit the transcriptional hallmarks of a stress response: downregulation of cytoplasmic ribosomes and upregulation of the environmental stress response genes. Furthermore, the cells from the slow-growing subpopulation express more transcripts from the 2-micron plasmid, transposons, endogenous virus-like RNAs, possibly as a means to increase diversity^38^; alternatively, the 2-micron plasmid and viruses may be trying to ‘jump ship’ from the stressed cell, similar to stress-induced herpes outbreaks^39^,^40^. Cells in the slow-growing subpopulation also express more of their genome, more novel and antisense transcripts, and there is more nucleotide diversity within each transcript, likely due to a decrease in transcription fidelity. Thus, the slow-growing subpopulation shows increased transcriptional diversity throughout the genome and may increase genic diversity to deal with future, unforeseen stresses.

While isogenic cells in the same environment are known to grow and divide at different rates, the intrinsic and extrinsic factors that cause cells to become slow or fast growing are not well understood. At the population level, stress reduces growth rate, and the transcriptional response to stress is highly stochastic^41^. Our results show that the growth rate response to stress is also highly heterogeneous. The relationship between stochastic differences in the transcriptional response to stress and growth rate within that stress remains to be elucidated.

Furthermore, cells are exposed to mild stresses growing in minimal media; cellular metabolism produces reactive oxygen species, and wild-type cells exhibit spontaneous Rad52 DNA damage foci^42^. We found that cells with Rad52–GFP foci gave rise to slow-growing microcolonies and that addition of the antioxidant vitamin C reduced the fraction of slow-growing cells. These results suggest that DNA damage or oxidative stress may cause cells to switch into a slow-growing state. The cellular growth rate response to some stresses is far noisier than for other stresses; growth in 2 M sorbitol produces the same modal microcolony growth rate as growth in 1 mM CoCl_2_, but the growth rate distribution in CoCl_2_ is significantly wider. Thus, certain, but not all types of intrinsic or extrinsic stress may cause cells to switch into a slow growth state. If this slow growth state is stress resistant, as results in prokaryotes^4^ and yeast^16^ suggest that it is, then this may be a bet-hedging mechanism in which small amounts of stress lead to slow growth and subsequent stress resistance of a subset of the population, while the majority of the population continues to grow quickly at the cost of remaining stress sensitive.

Stress and slow growth may increase both transposition^14^,^15^ and the DNA mutation rate^43^,^44^, and the cell is capable of altering the fidelity of both DNA^30^ and RNA^27^ polymerases. Because the growth rate within a population under stress is highly non-uniform, the potential for heritable changes may be different among cells within a population, in a manner that depends on the growth rate of individual cells. Therefore, a complete understanding of the way in which organisms evolve will require the continued development of novel methods for isolating and characterizing subpopulations that differ in dynamic phenotypes.

## Methods

### Yeast strains and media

All yeast strains are derived from BY4741 and were generated by homologous integration of PCR products. For all experiments, yeast were grown in Synthetic Complete media with 2% Dextrose (SCD).

### Overview of the FitFlow method

During budding, a chitin ring is formed in the cell wall at the bud neck between the mother and daughter. Following cytokinesis, bud neck contraction ends with the formation of a chitin septum that divides the mother and daughter. Both mother and daughter then build cell walls around this chitin septum. Finally, the chitin septum is degraded by chitinase, and the two cells separate^18^,^45^. In the absence of chitinase, the cell walls between the mother and daughter remain continuous. Because the cell wall consists crosslinked chitin, mannoproteins and glucans (polysaccharides), the mother and daughter remain attached to each other by covalent bonds. The cts1 deletion does not affect the HTA2–GFP fluorescence distribution, the ura3::TEFpr-mCherry distribution or the growth rate (Supplementary Fig. 18).

To collect a large subpopulation of cells with the same genotype and environment, but with different microcolony growth rates, an HTA2–GFP cts*1*Δ strain was grown overnight so that it reached an OD of 0.4 in the morning. Cells were then placed in a room temperature water bath and sonicated for 10 s at an amplitude of 10 using a Branson Digital Sonifier. A total of 250,000 G1 cells were then isolated by sorting at room temperature on the Histone–GFP signal. Cells were then allowed to grow into microcolonies at 30 °C for 4 h (two population doublings). Approximately 2 ml of cells from a single tube were then placed on ice, into a prechilled BD FACSAria, and sorted for 30 min at 4 °C by Histone–GFP content per microcolony. Cells were sorted into three bins: the lowest 10%, the median 10% and the highest 10%. Cells were sorted into prechilled 1.5-ml eppendorf tubes containing 250 μl SCD each. At least 50,000 microcolonies were isolated in each bin. Cells were then transferred on ice to a centrifuge at 4° C, spun at 14,000 r.p.m. (20,817*g*) for 2 min, the media removed and the resulting cell pellet frozen at −80° C for RNA extraction. All bins were treated identically throughout the process. Cellular RNA was extracted using the Epicenter MasterPure RNA Purification Kit, and Illumina sequencing libraries were prepared using the Truseq Stranded mRNA kit and sequenced on a HiSeq2000 with at least 20,000,000 50-bp sequencing reads per bin.

### RNA-seq expression levels and RNA polymerase fidelity

RNA-seq data from all experiments were processed as follows. FASTQ files (SRAs: SRR636634, SRR636633, SRR636635, SRR636636 SRR636637, SRR636638, SRR636639 SRR636640, SRR636641, SRR636642, SRR636643, SRR636644, SRR636645, SRR636646, SRR636647, SRR636648, SRR636649 and SRR636650 for the stress experiments^32^, and SRR453566, SRR453567, SRR453568, SRR453569, SRR453570 and SRR453571 for the batch versus chemostat experiments^33^) were downloaded. FASTQ files from FitFlow experiments were downloaded from the CRG genomics facility.

We performed two different types of expression analysis: *de novo* transcriptome assembly to identify novel transcripts, and guided assembly to compute differential expression of annotated transcripts. For the latter, differential gene expression was performed using cufflinks^46^ (–max-bundle-frags 100,000,000–frag-bias-correct–multi-read-correct) and cuffdiff (–multi-read-correct), using the R64 release of the yeast genome with viral and 2-μM plasmid sequences added. The annotation file used was the R64 gff with viral and 2-μM plasmid sequences added.

To identify novel transcripts, cufflinks was used in unguided mode. The raw RNA-seq data were mapped to the reference genome R64 with bowtie^47^ using the default parameters. The mapped reads were assembled into transcripts using cufflinks, not using the reference annotations as a guide (paramters -u -b - -overlap-radius 30 - -min-isoform-fraction 0.01). The resulting unguided assembly was then compared with the reference annotations from SGD using cuffcompare, which determines how the assembled transcripts overlap the reference annotations. Cuffcompare sorts the assembled transcripts into 12 class codes depending on the intersection of each transcript and the reference annotations; class codes ‘u’ and ‘x’ designate transcripts that do not overlap any annotated features, and transcripts found overlapping an annotated feature on the opposite strand, respectively.

We found that the absolute number and location of identified antisense and novel transcripts is not very reproducible across biological replicates. As an alternative method, we combined sequencing reads from all data, and performed a single unguided assembly using cufflinks. The unguided assembly was then used as a guide to run cufflinks on each group individually. This pooling approach decreased the variation between assemblies due to cufflinks splitting a transcript or joining adjacent transcripts in one group and not in another. The guided assemblies of each group were then compared with the reference annotations with cuffcompare. This approach produced the same qualitative result: slow-growing populations show higher expression of more novel and antisense transcripts.

To measure differential RNA polymerase error rates, reads were trimmed to remove the first five bases, which are of low quality. The qualitative difference (slow growers have more mismatches) is insensitive many different types of read trimming and filtering, as all experiments were sequenced in the same lane and similar error profiles. Reads were then aligned using bwa-aln^48^ to the R64 release of the *Saccharomyces cerevisiae* genome with default parameters. Relative mRNA error rates in *C. elegans* were determined using SRAs SRR1560104 SRR1560105, SRR1560106 and SRR1560107 (ref. 34). Reads were processed identically except that the alignment was performed with bwa-mem. For both yeast and worm data, samtools^49^ mpileup (paramters -q 30 -d 100,000 -C50) was used to identify positions at which RNA-seq reads differed from the reference genome. The resulting mpileup files were then processed using a custom perl script to count the number of mismatches relative to the total number of mapped bases.

### Differential expression and Gene Onotology (GO) term analysis

The differential expression of manually selected groups of genes (for example, the DNA damage response genes) was obtained directly from the FPKM (fragments per kilobase of exon per million mapped reads) outputs given by cuffdiff, as described above. GO term analysis was performed using GOrilla^50^ using the default *P* value threshold of 10^−3^. For GO term analysis using GOrilla, genes were sorted by the log2(FPKM_fast/FPKM_slow) with the constant term 0.1 added to remove zeros.

### Computing subpopulation growth-correlated gene expression

To quantify for each gene to what extent it is upregulated in either subpopulation slow- or fast-growing cells, we computed the log2 ratio of fast versus slow bin FPKM obtained from RNA-seq. Thus, our growth-correlated expression metric is log_2_(FPKM_fast_)−log_2_(FPKM_slow_).

### Computing mean population growth-correlated gene expression

To quantify how gene expression changes with growth across environmentally imposed conditions, we downloaded the data from Brauer *et al*.^9^. This data comprises microarray measurements across 36 different continuous culture (chemostat) conditions. Their data are computed as the log_2_ ratio of the sample signal (spot intensity) over the signal in the reference channel (glucose-limited chemostat grown at a dilution rate of 0.25 h^−1^). Thus, we can write this as log_2_(*S*_ratio,sample_)=log_2_(*S*_sample_)−log_2_(*S*_ref_), where *S*_sample_ and *S*_ref_ are the microarray spot intensities of the sample and reference, respectively. To compute one measure of growth-related expression for each gene we computed a single expression value for fast growth and a second single expression value for slow growth. The former is composed of all conditions with a chemostst dilution time of 0.05 h^−1^. The latter is composed of all conditions with a chemostat dilution time of 0.3 h^−1^. We then take the log2 ratio of the fast over the slow values, thus:




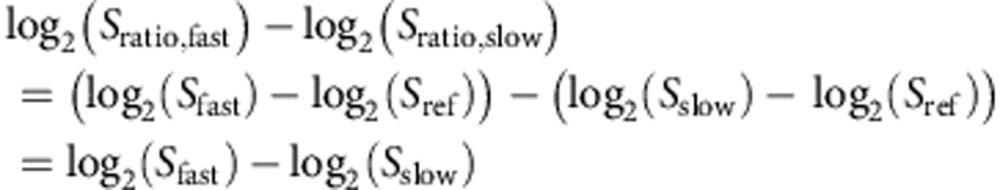




Where log_2_(*S*_ratio,fast_)and log_2_(*S*_ratio,slow_) are the average log2 ratios of the fast or slow samples over the reference signal, log_2_(*S*_fast_) and log_2_(*S*_slow_) are the average fast signal and slow signals.

### Genetic variability as a function of growth rate

Variation at each position in the transcriptome was determined using samtools mpileup. The genetic variability of the transcriptome was measured by computing, at each position in the genome, the total number of RNA-seq reads, and the number of reads that differ from the reference genome. Positions in which more than half the reads differ from the reference genome were discarded, under the assumption that these represent genomic differences between the experimental strain and the reference strain. Because errors are rare compared with the coverage of the genome, the error rate at any one position cannot be measured. Therefore, the transcriptome-wide or transcript-wide error rate is the total number of mismatches at all positions, divided by the total number of reads at all positions. To calculate the per-expression level error rate, positions were grouped by the total number of reads, and binned into groups of at least 100,000 different genomic positions with identical coverage. Because the dominant source of differences between RNA-seq data and the reference is technical (for example, sequencing errors, PCR and reverse transcription errors), and the frequency of these errors differs between labs, for each set of data we calculate the average error rate across all experiments performed by that lab. We then compute the relative increase or decrease in error rate for within each experiment.

### Microscopy

Microscopy was performed similar to that of Levy *et al*.^16^. Before each experiment, a 96-well glass bottom plate (Brooks Life Science Systems, MGB096-1-2-LG-L) was coated with 200 μl of 200 μg ml^−1^ concanavalin A (Sigma, type IV, Product # C2010) for 16 h at 37 °C. The plates were then washed with water and allowed to dry for at least 24 h at 4 °C.

Before microscopy, cells were grown overnight in 150 μl SCD to saturation, diluted 1:50 and grown overnight a second time. For experiments in which stress or drug was used, cells were grown for at least 24 h in the condition in which they were measured using microscopy. On the day of the microscopy experiment, cells were diluted 1:50 into 150 μl SCD (+drug/stress). Cells were grown for 4 h, shaking, at 30° C, and then diluted to an initial OD_600_ of 0.0007, and 80 μl of cells were added into 320 μl of media in a glass-bottomed microscopy plate, resulting in an initial OD_600_ of 0.00014. The plate was then sealed with PCR film, centrifuged and imaged for at least 12 h at 30 °C using an ImageXpress Micro (Molecular Devices) with a 10 × objective.

Microcolony segmentation of bright-field images was performed using custom perl scripts. Bright-field images were analysed in two steps. First, for each image the mean pixel intensity and s.d. were calculated, which allowed identification of the brightest and the darkest pixels of the image. Pixels with intensities greater than mean+2.5 × s.d. were considered as the brightest pixels of the image, whereas the pixels with intensities lower than mean−2.5 × s.d. were considered to be the darkest pixels. On top of that, Sobel edge detection was used to identify drastic change in pixel intensity in the image that happens at the yeast cell boundary. The pixels satisfying at least one of the above criteria were clustered and centroids for all clusters were calculated. In the second step, the centroids of clusters were computationally aligned across consecutive time points. Clusters showing at least doubling of area over the whole period of observation were considered as yeast microcolonies. If two clusters touched each other during our observation, they were only followed until the time point at which they touched. The resulting raw data consist of area measurements for each microcolony, and the time associated with each measurement.

Images in the fluorescent channel were processed to measure intensity of GFP signal. For each image, mean fluorescence intensity and the corresponding s.d. were calculated. Only pixels with intensities greater than mean+2 × s.d. were considered in our analysis. In the next step, the fluorescent pixels of interest were computationally superimposed with the bright-field images for calculation of maximum GFP intensity in a microcolony, which was calculated as the maximum fluorescent pixel intensity within the microcolony area. The resulting raw data consist of the sum, average and maximum GFP pixel intensity in each microcolony at each time point.

To calculate microcolony growth rates independent of both lag and competition for resources as the cell density increases, we use the maximum microcolony growth rate. Microcolony areas were natural-log transformed and area regressed against time. The growth rate for each microcolony is the maximum slope that spans at least three time points (180 min) with an *R*^2^≥0.95.

To calculate the fraction of slow-growing microcolonies, a tangent line is fit to the point of maximal slope in the cumulate distribution of the microcolony growth rates. In a uniform distribution, this line will explain 100% of the data. In a normal distribution, this line will explain most of the data, and the unexplained data will be symmetric in the bottom left and top right of the cdf. The fraction of slow microcolonies is unexplained slow−unexplained fast, or zero if this value is negative.

### Future adaptation of FitFlow for metazoans and bacteria

FitFlow relies on creating microcolonies of cells in which all cells in the microcolony are descended from a single cell. This method could be applied in bacteria using the drug cephalexin or FtsZ mutants, both of which cause *E. coli* to form chains^51^. It is more difficult to force mammalian cells to remain covalently bound after cytokinesis. However, this is not a requirement. A DNA dye pulse-chase or histone–GFP photoconversion^52^ would permit cells to be sorted by the number of divisions. In our experience with yeast, the Histone–GFP has a far narrower distribution than carboxyfluorescein succinimidyl ester, which is often used to determine the number of divisions cells have undergone. While it seems like an easy experiment to stain cell lines with carboxyfluorescein succinimidyl ester or a DNA dye, and, after several divisions, sort and sequence, to the best of our knowledge, no one has performed this experiment. This would enable FitFlow to be applied to mammalian cells.

## Additional information

**How to cite this article:** van Dijk, D. *et al*. Slow-growing cells within isogenic populations have increased RNA polymerase error rates and DNA damage. *Nat. Commun*. 6:7972 doi: 10.1038/ncomms8972 (2015).

## Supplementary Material

Supplementary Information Supplementary Figures 1-20

Supplementary Data 1 GO-term enrichment analysis for subpopulation and mean population growth-correlated expression. GOrilla GO-term analysis of one quadrant of subpopulation versus mean population growth correlated expression (e.g. Fig.3b). Quadrants are NW, NE, SE and SW representing top left, top right, bottom right and bottom left quadrants, thus giving gene sets for which subpopulation and mean population values are correlated (NE and SW) or anti-correlated (NW and SE). For each quadrant both ranked (sorted by distance from Y=X or Y=-X) and unranked (versus background) analysis was performed.

## References

[b1] Orr H. A. Fitness and its role in evolutionary genetics. Nat. Rev. Genet. 10, 531–539 (2009). 19546856 10.1038/nrg2603 PMC2753274

[b2] Kiviet D. J. . Stochasticity of metabolism and growth at the single-cell level. Nature 514, 376–379 (2014). 25186725 10.1038/nature13582

[b3] Wakamoto Y. . Dynamic persistence of antibiotic-stressed mycobacteria. Science 339, 91–95 (2013). 23288538 10.1126/science.1229858

[b4] Balaban N. Q., Gerdes K., Lewis K. & McKinney J. D. A problem of persistence: still more questions than answers? Nat. Rev. Microbiol. 11, 587–591 (2013). 24020075 10.1038/nrmicro3076

[b5] Fridman O., Goldberg A., Ronin I., Shoresh N. & Balaban N. Q. Optimization of lag time underlies antibiotic tolerance in evolved bacterial populations. Nature 513, 418–421 (2014). 25043002 10.1038/nature13469

[b6] Gupta P. B. . Stochastic state transitions give rise to phenotypic equilibrium in populations of cancer cells. Cell 146, 633–644 (2011). 21854987 10.1016/j.cell.2011.07.026

[b7] Brown R., Curry E., Magnani L., Wilhelm-Benartzi C. S. & Borley J. Poised epigenetic states and acquired drug resistance in cancer. Nat. Rev. Cancer 14, 747–753 (2014). 25253389 10.1038/nrc3819

[b8] Marusyk A., Almendro V. & Polyak K. Intra-tumour heterogeneity: a looking glass for cancer? Nat. Rev. Cancer 12, 323–334 (2012). 22513401 10.1038/nrc3261

[b9] Brauer M. J. . Coordination of growth rate, cell cycle, stress response, and metabolic activity in yeast. Mol. Biol. Cell 19, 352–367 (2008). 17959824 10.1091/mbc.E07-08-0779 PMC2174172

[b10] Kemmeren P. . Large-scale genetic perturbations reveal regulatory networks and an abundance of gene-specific repressors. Cell 157, 740–752. 24766815 10.1016/j.cell.2014.02.054

[b11] O’Duibhir E. . Cell cycle population effects in perturbation studies. Mol. Syst. Biol. 10, 732–732 (2014). 24952590 10.15252/msb.20145172 PMC4265054

[b12] Ihssen J. & Egli T. Global physiological analysis of carbon- and energy-limited growing *Escherichia coli* confirms a high degree of catabolic flexibility and preparedness for mixed substrate utilization. Environ. Microbiol. 7, 1568–1581 (2005). 16156730 10.1111/j.1462-2920.2005.00846.x

[b13] Ihssen J. & Egli T. Specific growth rate and not cell density controls the general stress response in *Escherichia coli*. Microbiology 150, 1637–1648 (2004). 15184550 10.1099/mic.0.26849-0

[b14] Lubeck E. & Cai L. Single-cell systems biology by super-resolution imaging and combinatorial labeling. Nat. Methods 9, 743–748 (2012). 22660740 10.1038/nmeth.2069 PMC3418883

[b15] Elowitz M. B., Levine A. J., Siggia E. D. & Swain P. S. Stochastic gene expression in a single cell. Science 297, 1183–1186 (2002). 12183631 10.1126/science.1070919

[b16] Levy S. F., Ziv N. & Siegal M. L. Bet hedging in yeast by heterogeneous, age-correlated expression of a stress protectant. PLoS Biol. 10, e1001325–e1001325 (2012). 22589700 10.1371/journal.pbio.1001325 PMC3348152

[b17] Ziv N., Siegal M. L. & Gresham D. Genetic and non-genetic determinants of cell-growth variation assessed by high-throughput microscopy. Mol. Biol. Evol. 30, 2568–2578 (2013). 23938868 10.1093/molbev/mst138 PMC3840306

[b18] Cabib E. & Arroyo J. How carbohydrates sculpt cells: chemical control of morphogenesis in the yeast cell wall. Nat. Rev. Microbiol. 11, 648–655 (2013). 23949603 10.1038/nrmicro3090

[b19] Neymotin B., Athanasiadou R. & Gresham D. Determination of in vivo RNA kinetics using RATE-seq. RNA 20, 1645–1652 (2014). 25161313 10.1261/rna.045104.114 PMC4174445

[b20] Hillenmeyer M. E. . The chemical genomic portrait of yeast: uncovering a phenotype for all genes. Science 320, 362–365 (2008). 18420932 10.1126/science.1150021 PMC2794835

[b21] Roberts G. G. & Hudson A. P. Transcriptome profiling of *Saccharomyces cerevisiae* during a transition from fermentative to glycerol-based respiratory growth reveals extensive metabolic and structural remodeling. Mol. Genet. Genomics 276, 170–186 (2006). 16741729 10.1007/s00438-006-0133-9

[b22] Ebina H. & Levin H. L. Stress management: how cells take control of their transposons. Mol. Cell 27, 180–181 (2007). 17643368 10.1016/j.molcel.2007.07.004

[b23] Feng G., Leem Y. E. & Levin H. L. Transposon integration enhances expression of stress response genes. Nucleic Acids Res. 41, 775–789 (2012). 23193295 10.1093/nar/gks1185 PMC3553992

[b24] Galhardo R. S., Hastings P. J. & Rosenberg S. M. Mutation as a stress response and the regulation of evolvability. Crit. Rev. Biochem. Mol. Biol. 42, 399–435 (2007). 17917874 10.1080/10409230701648502 PMC3319127

[b25] Hastings P. J., Slack A., Petrosino J. F. & Rosenberg S. M. Adaptive amplification and point mutation are independent mechanisms: evidence for various stress-inducible mutation mechanisms. PLoS Biol. 2, e399–e399 (2004). 15550983 10.1371/journal.pbio.0020399 PMC529313

[b26] Gasch A. P. . Genomic expression responses to DNA-damaging agents and the regulatory role of the yeast ATR homolog Mec1p. Mol. Biol. Cell 12, 2987–3003 (2001). 11598186 10.1091/mbc.12.10.2987 PMC60150

[b27] Fikus M. U. . The product of the DNA damage-inducible gene of *Saccharomyces cerevisiae*, DIN7, specifically functions in mitochondria. Genetics 154, 73–81 (2000). 10628970 10.1093/genetics/154.1.73 PMC1460913

[b28] Lisby M., Mortensen U. H. & Rothstein R. Colocalization of multiple DNA double-strand breaks at a single Rad52 repair centre. Nat. Cell Biol. 5, 572–577 (2003). 12766777 10.1038/ncb997

[b29] Cooke M. S., Evans M. D., Dizdaroglu M. & Lunec J. Oxidative DNA damage: mechanisms, mutation, and disease. FASEB J. 17, 1195–1214 (2003). 12832285 10.1096/fj.02-0752rev

[b30] McClintock B. The significance of responses of the genome to challenge. Science 226, 792–801 (1984). 15739260 10.1126/science.15739260

[b31] Gordon A. J. E., Satory D., Halliday J. A. & Herman C. Heritable change caused by transient transcription errors. PLoS Genet. 9, e1003595–e1003595 (2013). 23825966 10.1371/journal.pgen.1003595 PMC3694819

[b32] Baker L. A. . The yeast Snt2 protein coordinates the transcriptional response to hydrogen peroxide-mediated oxidative stress. Mol. Cell. Biol. 33, 3735–3748 (2013). 23878396 10.1128/MCB.00025-13 PMC3811877

[b33] Nookaew I. . A comprehensive comparison of RNA-Seq-based transcriptome analysis from reads to differential gene expression and cross-comparison with microarrays: a case study in *Saccharomyces cerevisiae*. Nucleic Acids Res. 40, 10084–10097 (2012). 22965124 10.1093/nar/gks804 PMC3488244

[b34] Schieber M. & Chandel N. S. TOR signaling couples oxygen sensing to lifespan in *C. elegans*. Cell Rep. 9, 9–15 (2014). 25284791 10.1016/j.celrep.2014.08.075 PMC4194168

[b35] Lang G. I. & Murray A. W. Estimating the per-base-pair mutation rate in the yeast *Saccharomyces cerevisiae*. Genetics 178, 67–82 (2008). 18202359 10.1534/genetics.107.071506 PMC2206112

[b36] Nesser N. K., Peterson D. O. & Hawley D. K. RNA polymerase II subunit Rpb9 is important for transcriptional fidelity in vivo. Proc. Natl Acad. Sci. USA 103, 3268–3273 (2006). 16492753 10.1073/pnas.0511330103 PMC1413937

[b37] Gout J.-F., Thomas W. K., Smith Z., Okamoto K. & Lynch M. Large-scale detection of in vivo transcription errors. Proc. Natl Acad. Sci. USA 110, 18584–18589 (2013). 24167253 10.1073/pnas.1309843110 PMC3832031

[b38] Koonin E. V. & Wolf Y. I. Evolution of microbes and viruses: a paradigm shift in evolutionary biology? Front. Cell. Infect. Microbiol. 2, 119–119 (2012). 22993722 10.3389/fcimb.2012.00119 PMC3440604

[b39] Kushnir A. S., Davido D. J. & Schaffer P. A. Role of nuclear factor Y in stress-induced activation of the herpes simplex virus type 1 ICP0 promoter. J. Virol. 84, 188–200 (2010). 19828605 10.1128/JVI.01377-09 PMC2798407

[b40] Volcy K. & Fraser N. W. DNA damage promotes herpes simplex virus-1 protein expression in a neuroblastoma cell line. J. Neurovirol. 19, 57–64 (2013). 23354549 10.1007/s13365-012-0140-z PMC3572510

[b41] Pelet S. . Transient activation of the HOG MAPK pathway regulates bimodal gene expression. Science 332, 732–735 (2011). 21551064 10.1126/science.1198851

[b42] Alvaro D., Lisby M. & Rothstein R. Genome-wide analysis of Rad52 foci reveals diverse mechanisms impacting recombination. PLoS Genet. 3, e228 (2007). 18085829 10.1371/journal.pgen.0030228 PMC2134942

[b43] Shor E., Fox C. A. & Broach J. R. The yeast environmental stress response regulates mutagenesis induced by proteotoxic stress. PLoS Genet. 9, e1003680 (2013). 23935537 10.1371/journal.pgen.1003680 PMC3731204

[b44] Roth J. R., Kugelberg E., Reams A. B., Kofoid E. & Andersson D. I. Origin of mutations under selection: the adaptive mutation controversy. Annu. Rev. Microbiol. 60, 477–501 (2006). 16761951 10.1146/annurev.micro.60.080805.142045

[b45] Weiss E. L. Mitotic exit and separation of mother and daughter cells. Genetics 192, 1165–1202 (2012). 23212898 10.1534/genetics.112.145516 PMC3512134

[b46] Trapnell C. . Differential gene and transcript expression analysis of RNA-seq experiments with TopHat and Cufflinks. Nat Protoc 7, 562–578 (2012). 22383036 10.1038/nprot.2012.016 PMC3334321

[b47] Langmead B., Trapnell C., Pop M. & Salzberg S. L. Ultrafast and memory-efficient alignment of short DNA sequences to the human genome. Genome Biol. 10, R25 (2009). 19261174 10.1186/gb-2009-10-3-r25 PMC2690996

[b48] Li H. & Durbin R. Fast and accurate short read alignment with Burrows-Wheeler transform. Bioinformatics 25, 1754–1760 (2009). 19451168 10.1093/bioinformatics/btp324 PMC2705234

[b49] Li H. . The sequence alignment/map format and SAMtools. Bioinformatics 25, 2078–2079 (2009). 19505943 10.1093/bioinformatics/btp352 PMC2723002

[b50] Eden E., Navon R., Steinfeld I., Lipson D. & Yakhini Z. GOrilla: a tool for discovery and visualization of enriched GO terms in ranked gene lists. BMC Bioinformatics 10, 48 (2009). 19192299 10.1186/1471-2105-10-48 PMC2644678

[b51] Pogliano J., Pogliano K., Weiss D. S., Losick R. & Beckwith J. Inactivation of FtsI inhibits constriction of the FtsZ cytokinetic ring and delays the assembly of FtsZ rings at potential division sites. Proc. Natl Acad. Sci. USA 94, 559–564 (1997). 9012823 10.1073/pnas.94.2.559 PMC19552

[b52] Elowitz M. B., Surette M. G., Wolf P. E., Stock J. & Leibler S. Photoactivation turns green fluorescent protein red. Curr. Biol. 7, 809–812 (1997). 9368766 10.1016/s0960-9822(06)00342-3

[b53] Wapinski I., Pfeffer A., Friedman N. & Regev A. Natural history and evolutionary principles of gene duplication in fungi. Nature 449, 54–61 (2007). 17805289 10.1038/nature06107

