# Supplementary material for: Slow-growing cells within isogenic populations have increased RNA polymerase error rates and DNA damage
Source: Nat Commun. 2015 Aug 13;6:7972. doi: 10.1038/ncomms8972 (PMC4557116; doi:10.1038/ncomms8972)
Supplement: Supplementary Information — Supplementary Figures 1-20 [file ncomms8972-s1.pdf]

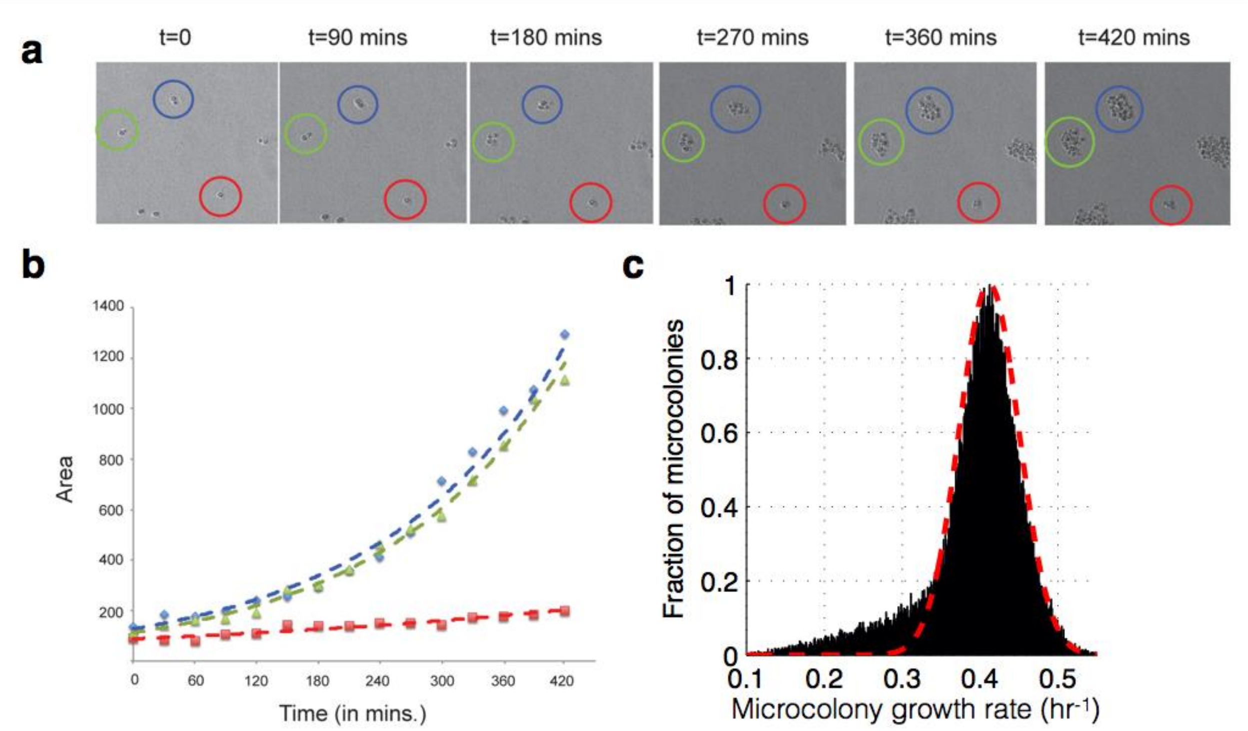

Supplementary Figure 1. An HTA2-GFP ura3::TEFpr-mCherry-URA3 $CTS1^{+}$ strain was grown to mid-log phase and measured using high-throughput timelapse microscopy. **(a,b)** A single slow-growing microcolony can be observed (red). **(c)** The distribution is not well fit by a normal distribution (dashed red line), but has a slow-growing tail.

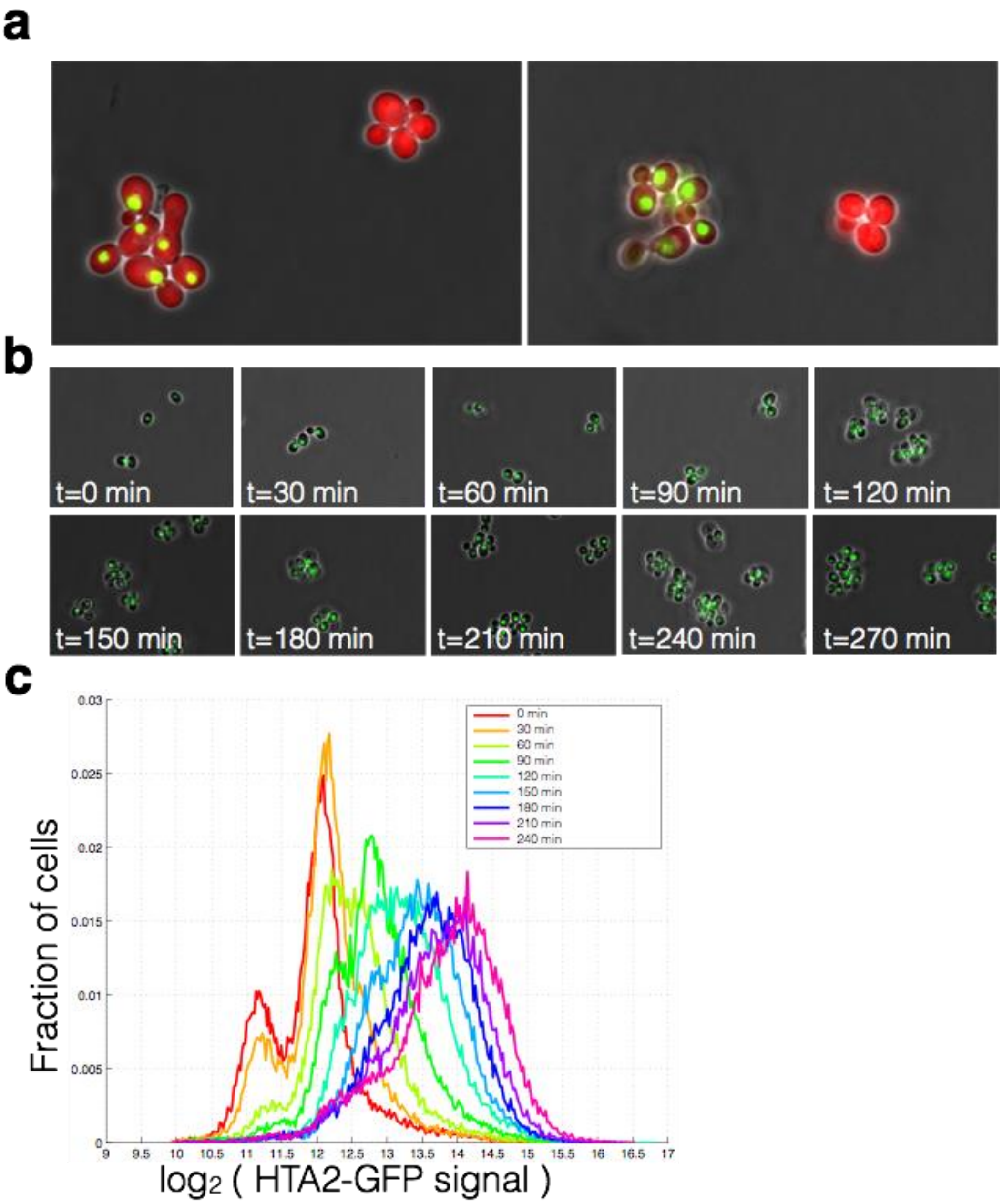

Supplementary Figure 2. (a) Only lineage related cells form clumps. HTA2-GFP cts1Δ ura3::TEFpr-mCherry-URA3 and cts1::TEFpr-mCherry-URA3 were sonicated separately, mixed in equal amounts, and grown together for five hours. Clumps consist of either entirely HTA2-GFP or of non-green cells. There are no clumps with mixed genotypes. **(b)** On average, clump size increases with time. HTA2-GFP cts1Δ cells were grown to mid-log phase, sonicated, and grown at 30C in SCD. **(c)** Sonication efficiently breaks apart clumps. Cells were grown to mid-log phase and sonicated as in (b), and samples were measured every 30 minutes by flow-cytometry. The distribution at t0 shows 1N and 2N peaks as are expected from a histone-GFP strain. The GFP signal per clump increases over time.

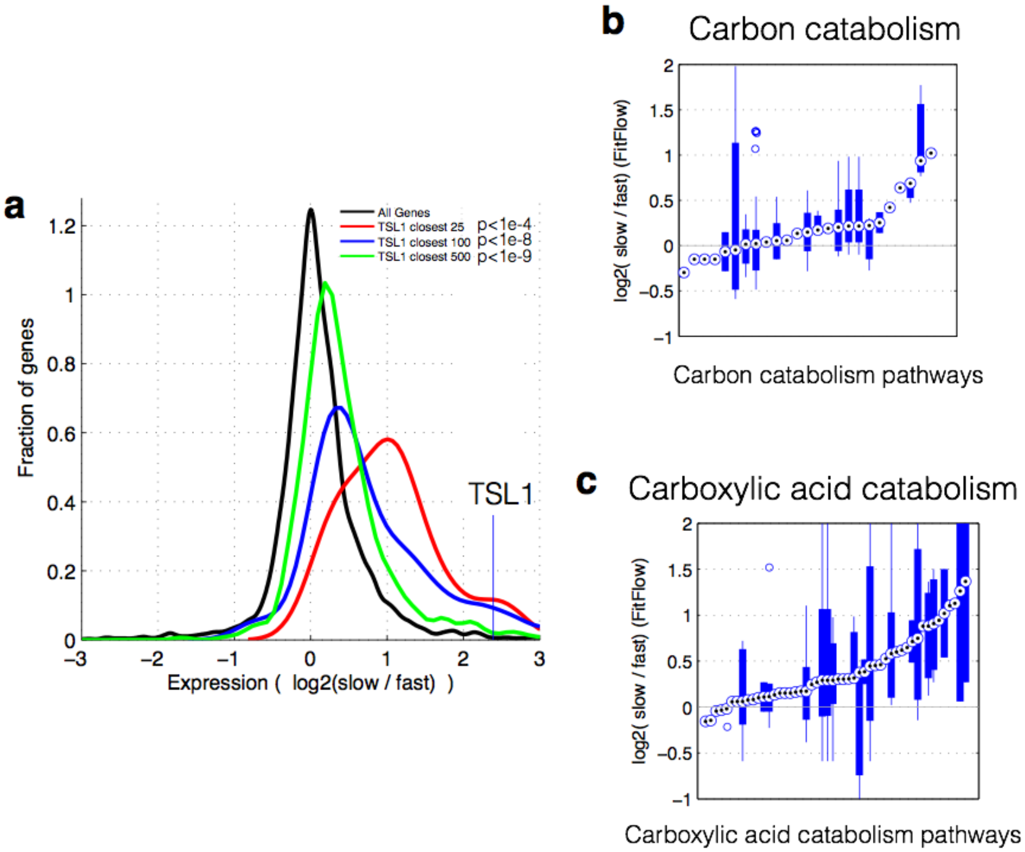

Supplementary Figure 3. The slow growing subpopulation transcriptional recapitulates what is known about slow growing yeast sub-populations, and slow growing *E coli* in a chemostat. **(a)** TSL1 and co-regulated transcripts are more highly expressed in the slow growing sub-population[16]. Levy *et al*. found that TSL1-GFP is more highly expressed in slow growing microcolonies. Consistent with these results, we find that TSL1 (blue vertical line), and genes that are co-regulated with TSL1 (red, blue & green distributions), are more highly expressed in the slow growing subpopulation according to FitFlow. Co-regulated genes were taken from SPELL[53]. The distribution of 'all genes' was used for t-tests to determine p-values. **(b)** Ihssen & Egli[12] showed that *E. coli* growing slowly in a chemostat express enzymes involved in the catabolism of a diverse range of carbon sources and carboxylic acids. The FitFlow yeast slow growing subpopulation has higher expression of pathways involved in the catabolism of alternative carbon sources and carboxylic acids. The log2(slow/fast) expression level for each gene in each pathway involved in (a) metabolism of different carbon sources and (b) metabolism of different amino acids and other carboxylic acids. Each pathway is shown as a single box in the boxplots. Pathways were obtained from SGD[54].

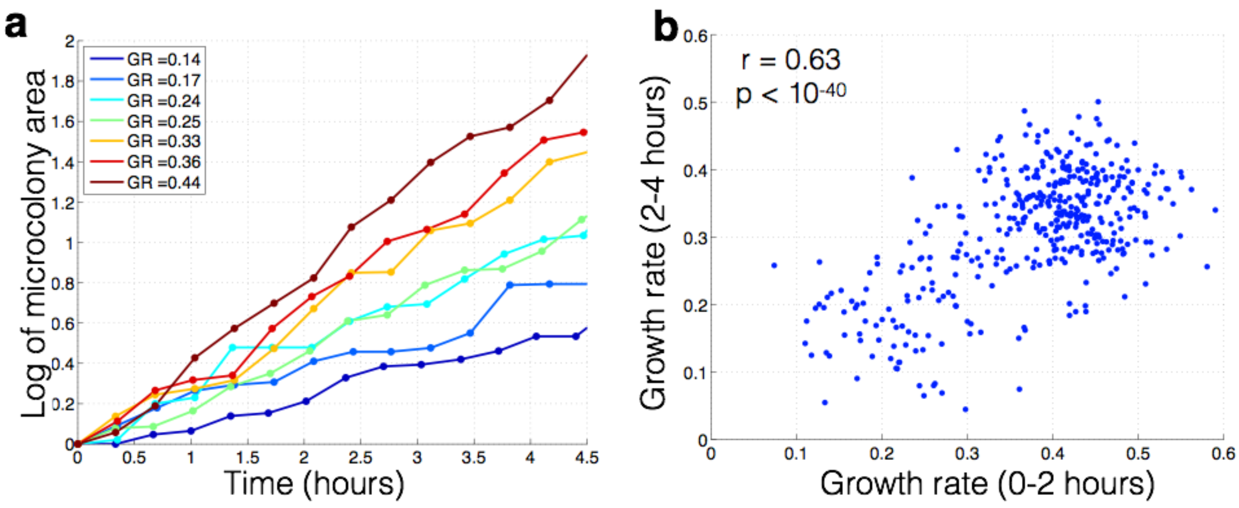

Supplementary Figure 4. Growth rate differences between microcolonies are heritable. Variability in microcolony growth rate was measured using time-lapse microscopy. **(a)** Growth rates (increase in area over time) of seven microcolonies. **(b)** For all (>1000) microcolonies, the growth rate during the first two hours of the experiment is highly correlated with the growth rate during the second two hours.

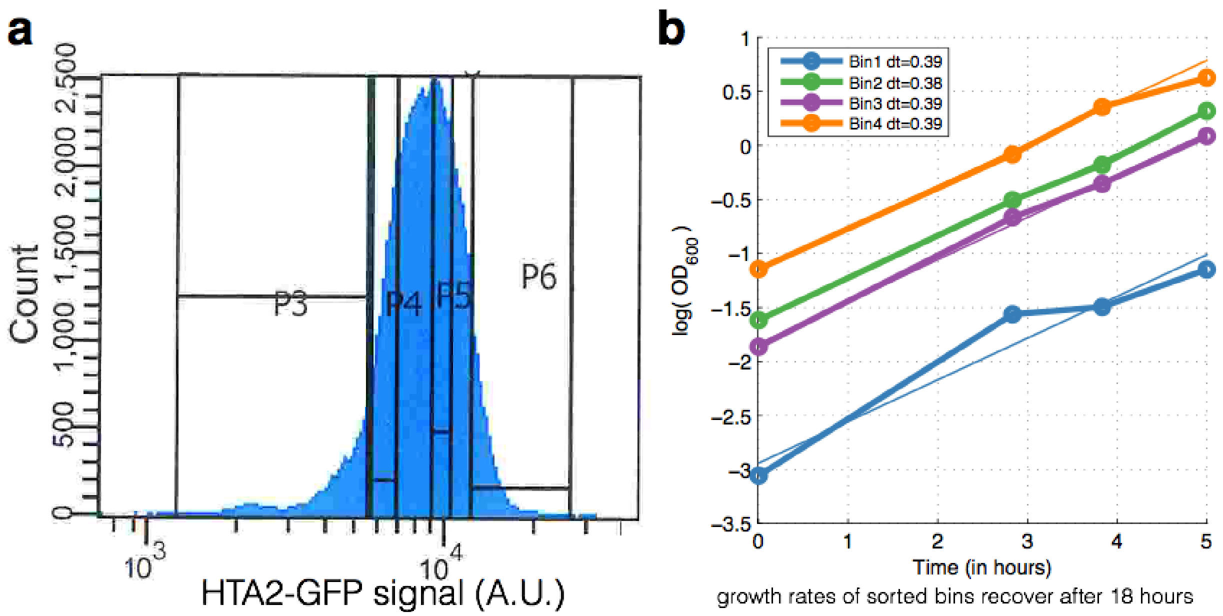

Supplementary Figure 5. The sorted slow subpopulation recovers the original population growth rate. The FITFLOW strain (Y40, HTA2-GFP TEFpr-mCherry, cts1::KanMX) was grown in SCD overnight to OD=0.1, G1 cells were sorted, grown for 3.5 hours at 30C in SCD. **(a)** Four bins of different HTA2-GFP/microcolony (8%, 14%, 19%, 8%) were sorted into 1.5ml eppendorf tubes at room temperature. 500ul of sorted cells were diluted into 6ml of SCD and grown at 30C. **(b)** Cell density measurements were started when the cultures had reached densities high enough to measure accurately.

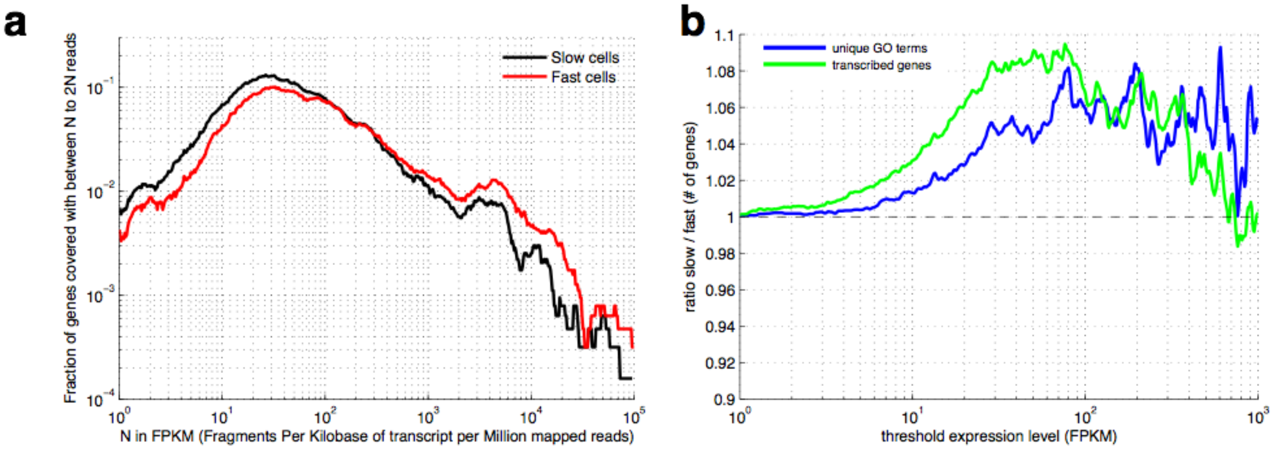

Supplementary Figure 6. Slow growing subpopulation express more genes at low expression, while fast growing cells express more highly expressed genes **(a)** Slow growing subpopulation have more genes with an expression level less than 100 FPKM, while fast growing cells have more genes with expression > 1000 FPKM (paired ks-test p=1.99*10^-28). **(b)** Slow growing subpopulation express more genes and unique GO terms (gene functions) even after correction for possible detection bias due to highly expressed genes. To correct for possible bias, reads from highly expressed (>1000 FPKM) genes were removed (the top 321 genes for fast, top 305 genes for slow), and expression levels recalculated for each gene. In addition, if this effect were due to increased ability to detect genes with low expression in stochastically slow cells (due to low ribosomal gene expression), the ratio of transcribed genes would be high at a cutoff (x-axis) of zero, and decrease from there. However, almost all transcripts are detected in both slow and fast cells, therefore the ratio (y-axis) is 1 for a cutoff less than 2 FPKM.

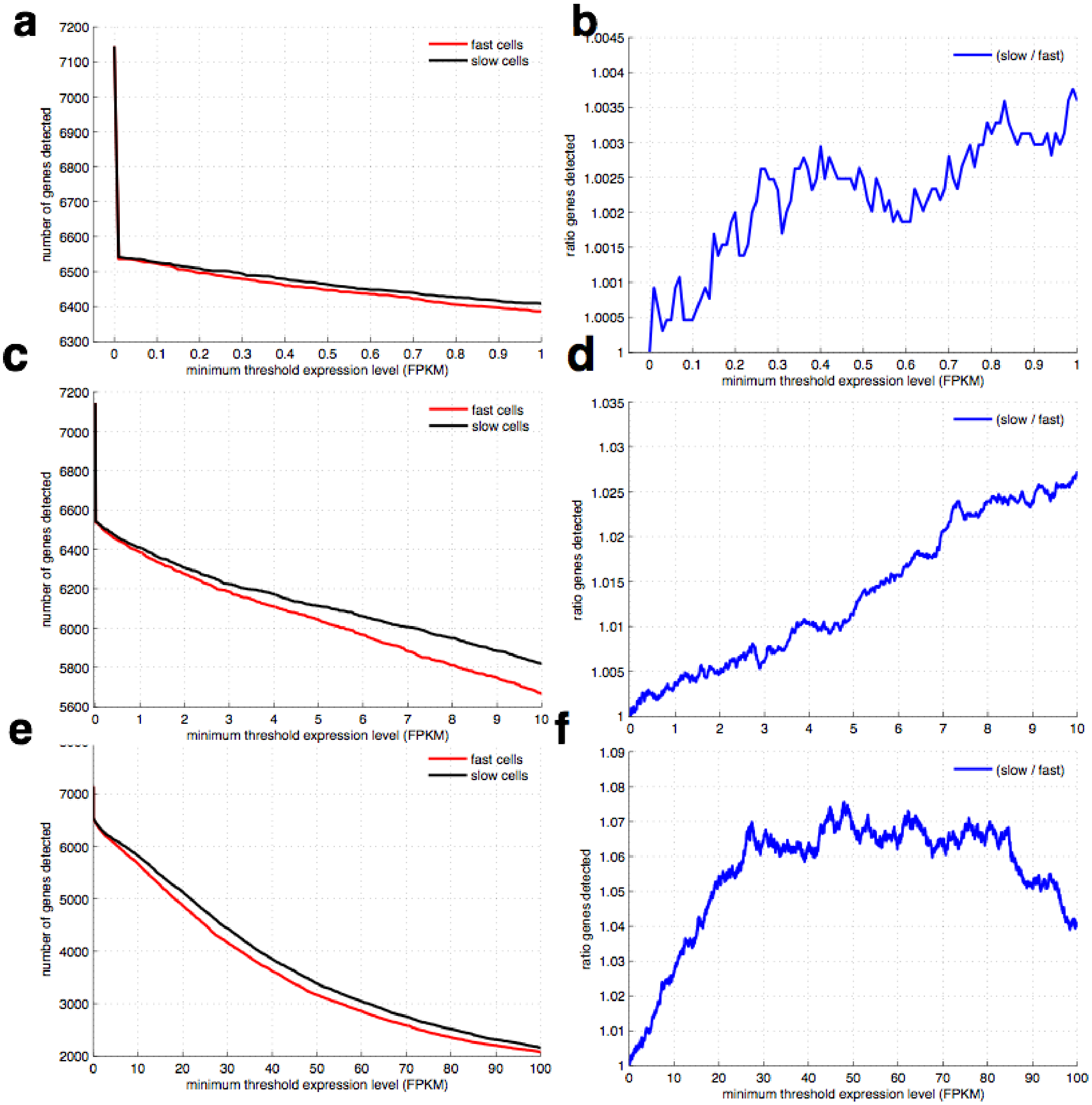

Supplementary Figure 7. Transcripts with very low expression levels are equally well detected in stochastically slow and fast growing cells. Shown are the number of detected genes with X or greater FPKM, for slow and fast growing cells (red and black lines), and the slow/fast ratio of this number (blue lines). For a threshold of 0 – 1 (very low expression or undetected), slow growing subpopulation have less than 0.3% more detected transcripts. For low to moderate levels of expression (~30 FPKM), slow growing subpopulation have 6% more detected transcripts. If the differences in the number of expressed transcripts were due to detection bias, the ratio (blue lines) would start high and decrease to a plateau.

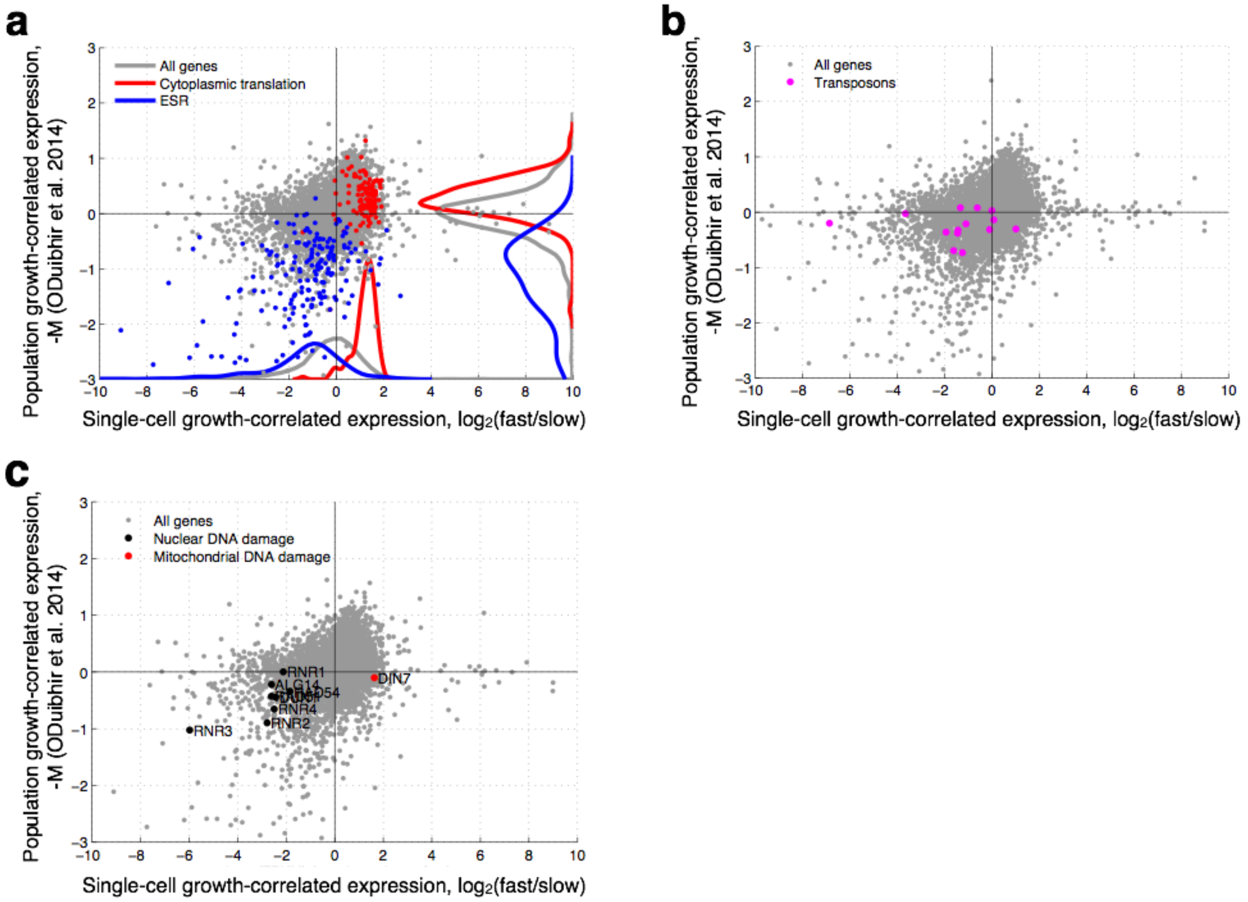

Supplementary Figure 8. RNA-seq data from stochastically different cells obtained by FitFlow (X-axis) are compared to microarray results from O'Duibhir et al. (Y-axis) in which growth rate were altered by knocking out genes. **(a)** cytoplasmic translation (Y-axis, paired t-test p=3.98*10^-16) and stress genes (Y-axis, paired t-test p=8.11*10^-87) and **(b)** transposons (Y-axis, paired t-test p=0.011) show similar results with data from O'Duibhir et al and from Brauer et al. **(c)** The nuclear DNA damage response genes are part of the slow growth signature of O'Duibhir et al. (Y-axis, paired t-test p=0.0017) but not of Brauer et al. (see Fig.3d).

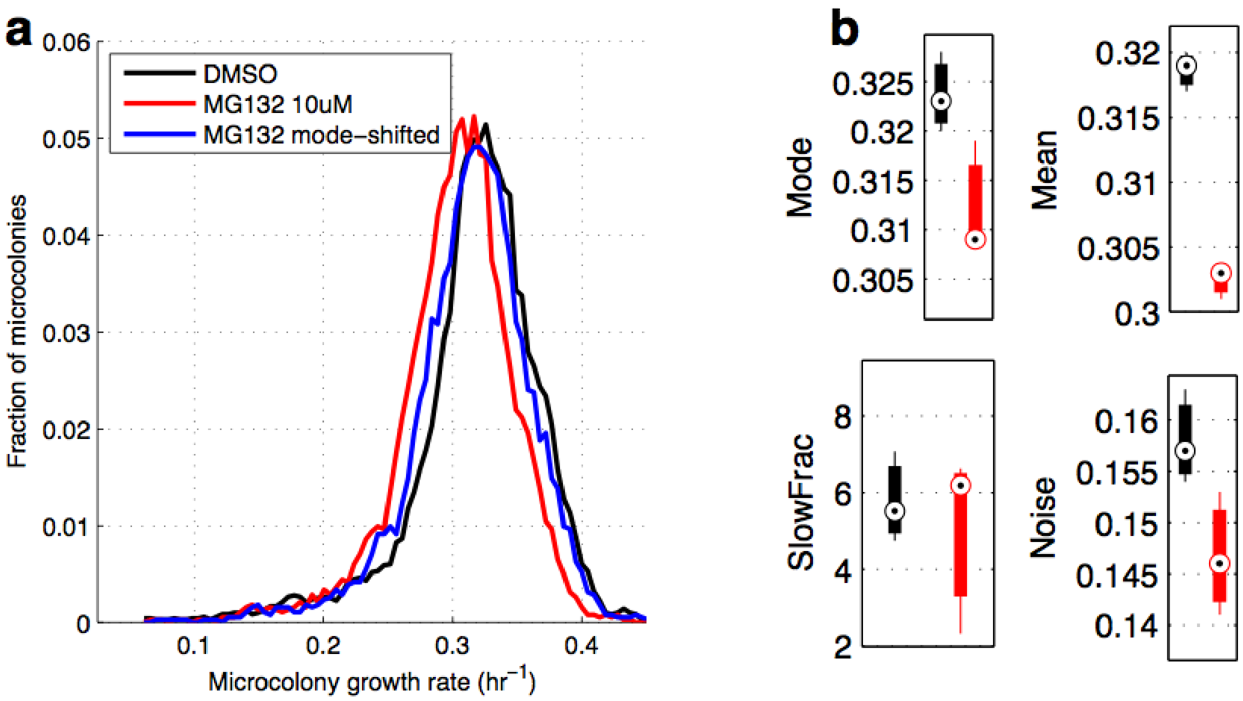

Supplementary Figure 9. The proteasome inhibitor MG132 reduces the growth rate of the fast growing cells more than the slow growing cells. A BY4741 pdr5::KanMX strain was grown in 0.024% DMSO or 0.024% DMSO + 10uM MG132. Pdr5 is a multi-drug transporter that pumps MG132 out of the cell; the knockout is necessary for inhibition. **(a)** Histograms of microcolony growth rates showing data from cells grown in MG132 (red) and the same distribution shifted so that the mode is the same as when cells are grown in DMSO (blue). Even after the mode-shift, the growth rate distribution of cells grown in MG132 is shifted to the left, suggesting that faster growing microcolonies are more affected than more slowly growing microcolonies (ks-test, $p<1e-4$). **(b)** MG132 decreases the mean population growth rate, and also decreases the noise (C.V.) of the distribution.

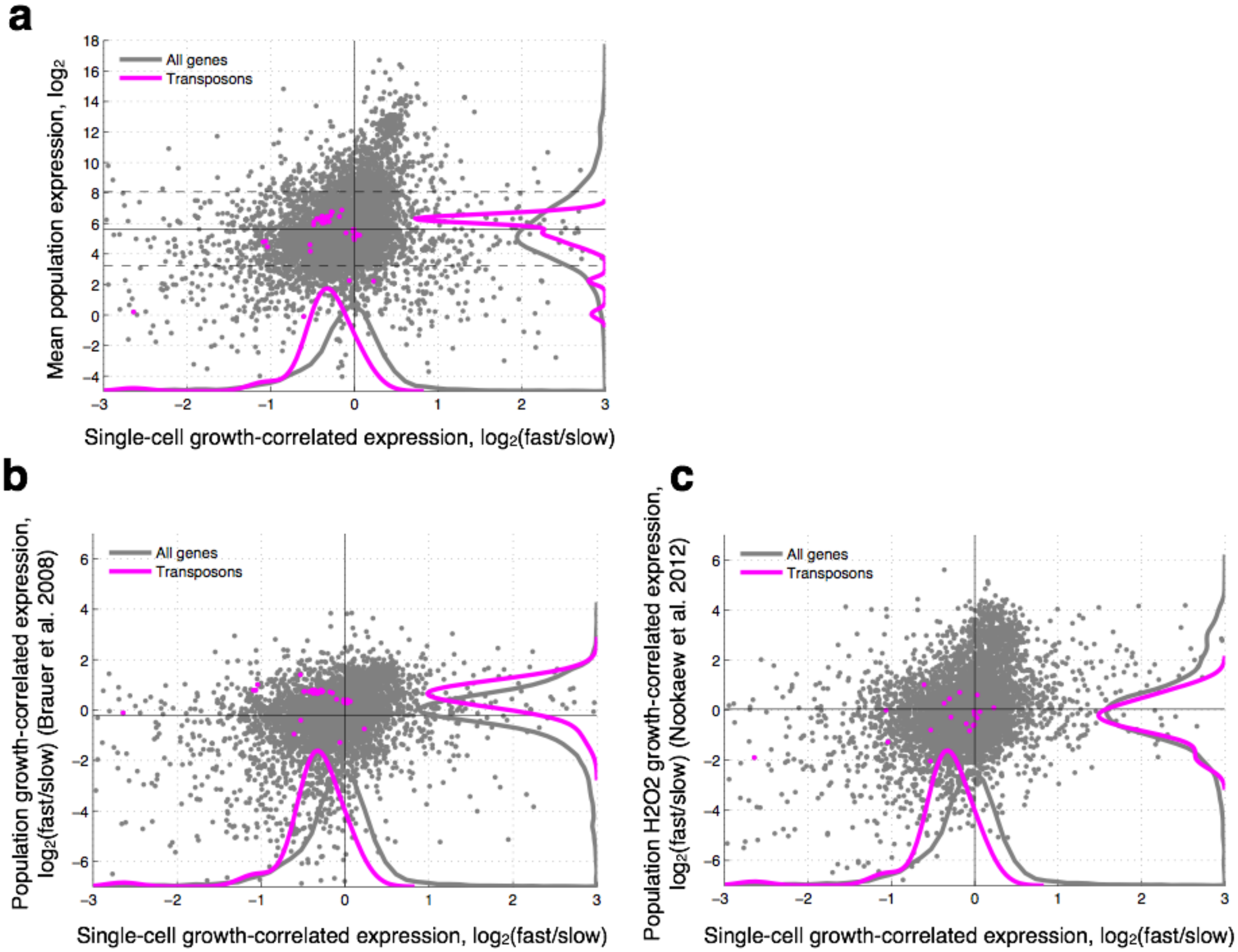

Supplementary Figure 10. Transposons are up-regulated in slow growing subpopulation, but show no significant correlation with (**a**) mean population expression (Y-axis, paired t-test p=0.78), mean population growth changes, both for growth changed in a chemostat **(b)**, Brauer et al. 2008, p=0.13) and growth changed as a result of H_2O_2 stress **(c)**, Baker et al. 2012, p=0.33).

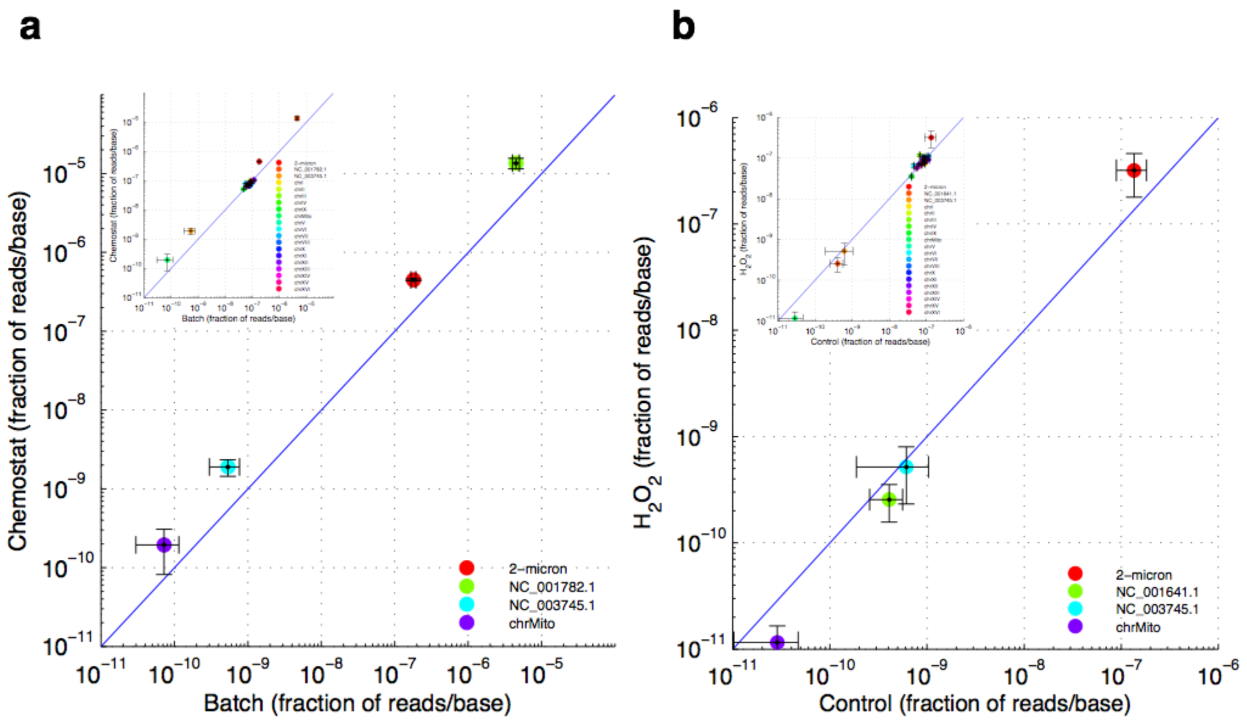

Supplementary Figure 11. Selfish DNA elements are more highly expressed in slow growing cells in a chemostat. Sequencing coverage (reads per nucleotide per sequenced base) for viruses (NC_*), the 2-micron plasmid, and the mitochondria. The inset shows the same calculation including the chromosomes. Error bars are standard deviation across three replicates for chemostat vs batch, and nine for H_2O_2 vs no stress. **(a)** Selfish genetic elements are upregulated in yeast slowly growing in a chemostat. **(b)** Genes on the 2uM plasmid are upregulated following thirty minutes in H_2O_2, while genes on the mitochondria are downregulated.

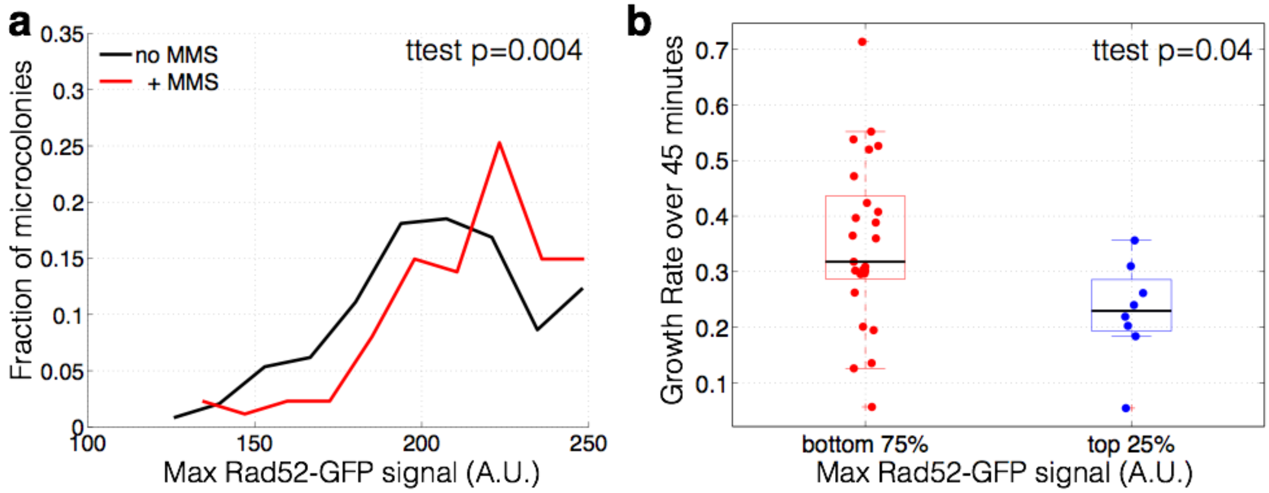

Supplementary Figure 12. **DNA damage can be measured by maximal Rad52-GFP intensity.** Rad52-GFP cells were grown to mid-log phase and imaged in brightfield and GFP every 45 minutes using time-lapse microscopy **(a)** MMS (1% final concentration) results in more cells having a brighter Rad52-GFP signal, suggesting that this is an accurate method for detecting DNA damage. **(b)** Cells with a high maximal Rad52-GFP signal grow more slowly over the subsequent 45 minutes, consistent with DNA damage resulting in slow growth. However, there is no difference in growth rate over two hours (t-test p=0.5).

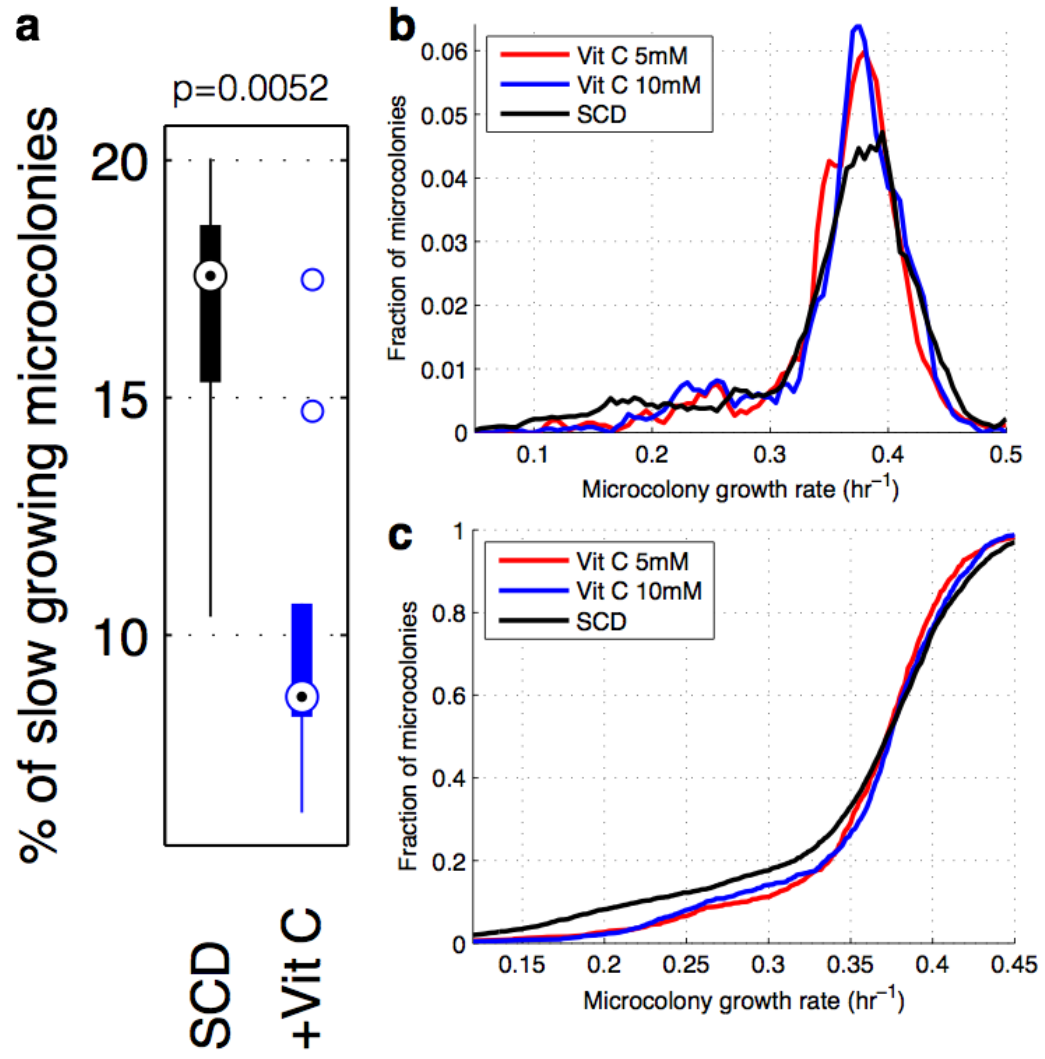

Supplementary Figure 13. Addition of an antioxidant reduced the fraction of slow growing microcolonies. Strain BY4741 were pre-grown in various concentrations of vitamin C (L-ascorbic acid) and microcolonies growth rates were measured using time-lapse microcopy. **(a)** Cells growing in vitamin C exhibit a significant reduction in the fraction of slow growing microcolonies (t-test, p=0.0052) **(b,c)** Three representative biological replicates.

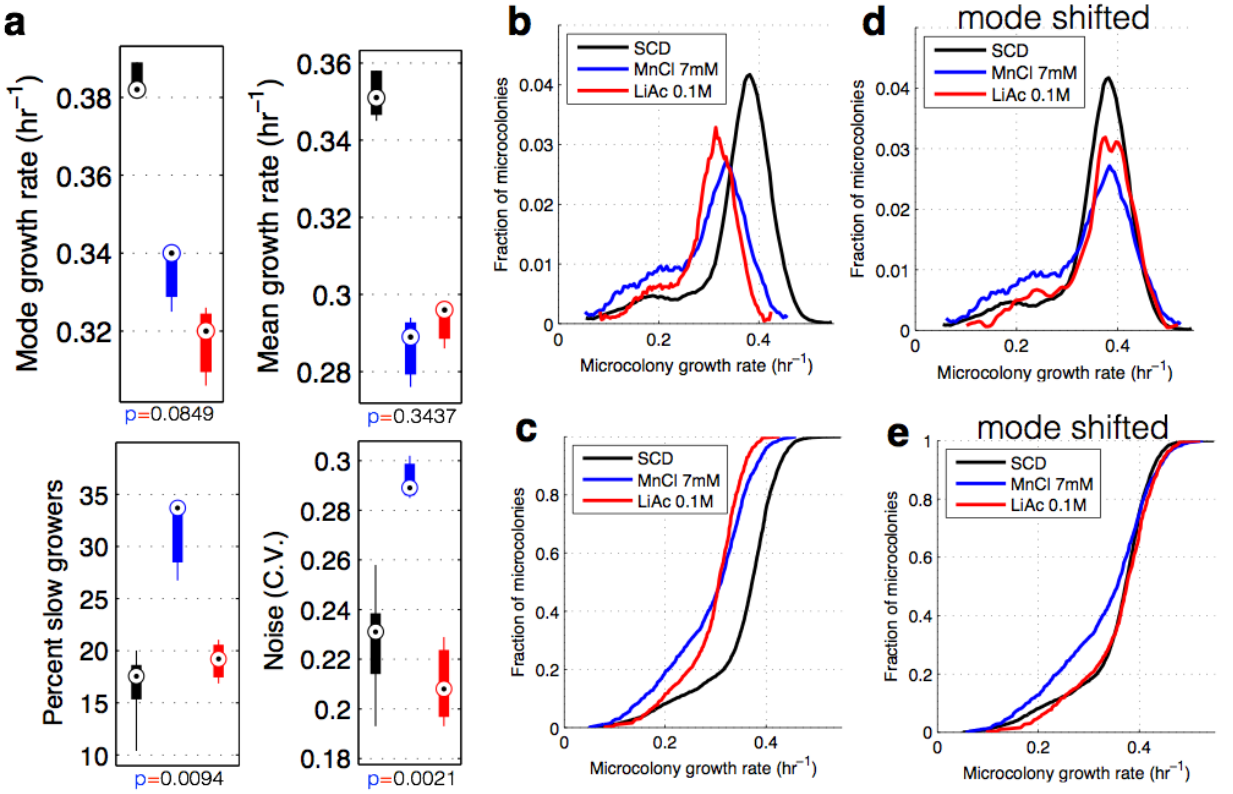

Supplementary Figure 14. Growth in the presence of MnCl results in a larger increase in the fraction of slow growing microcolonies than does growth in the presense of LiAc . Yeast were pre-grown in various concentrations of LiAc and MnCl and microcolonies growth rates were measured using time-lapse microcopy. Growth in 7mM MnCl and 100m LiAc results in the same mean and mode growth rate (t-test p=0.34, p=0.085) but very different distribution shapes.

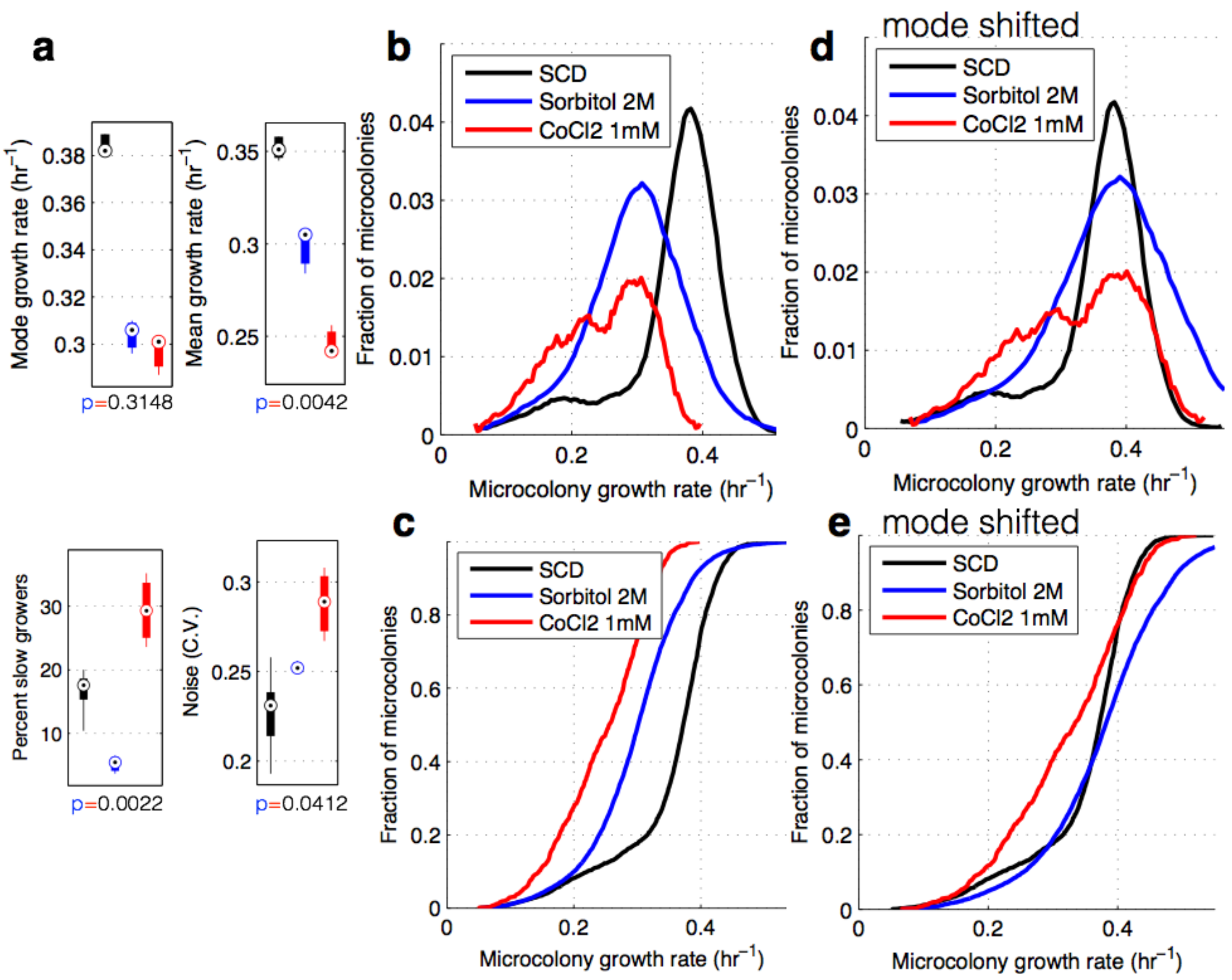

Supplementary Figure 15. Growth in the presence of $CoCl_2$ results in a larger increase in the fraction of slow growing microcolonies than does growth in the presence of sorbitol . Yeast were pre-grown in various concentrations of $CoCl_2$ and sorbitol and microcolonies growth rates were measured using time-lapse microcopy. Growth in 2M sorbitol and 1mM $CoCl_2$ results in the same mode growth rate (t-test p=0.31) but very different distribution shapes.

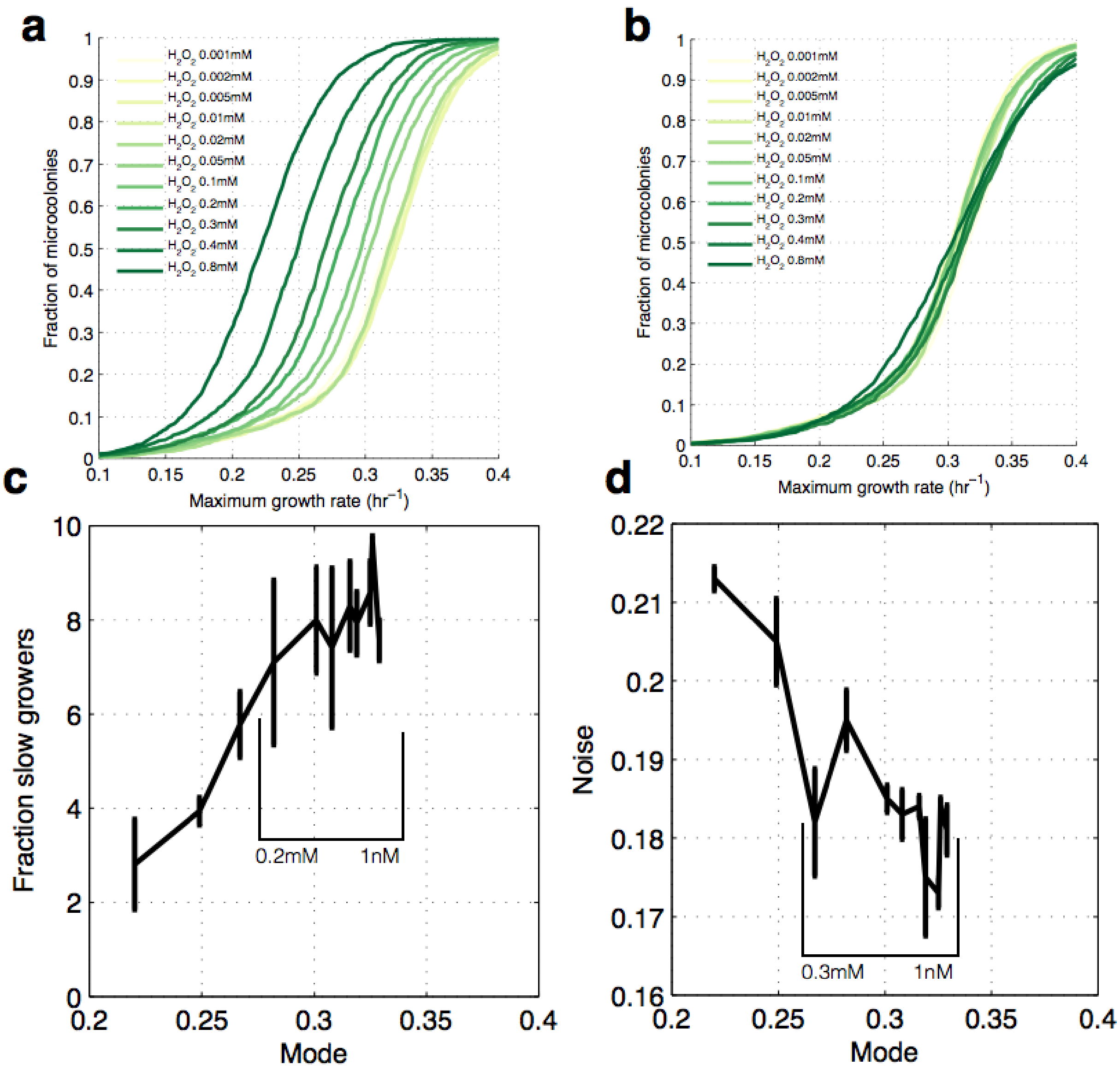

Supplementary Figure 16. Growth in the presence of H_2O_2 changes the mean without changing the distribution shape. Yeast were pre-grown in various concentrations of H_2O_2 and microcolonies growth rates were measured using time-lapse microcopy. Growth in up to 0.2mM H_2O_2 does not change the overall shape of the distribution or the fraction of slow growing microcolonies.

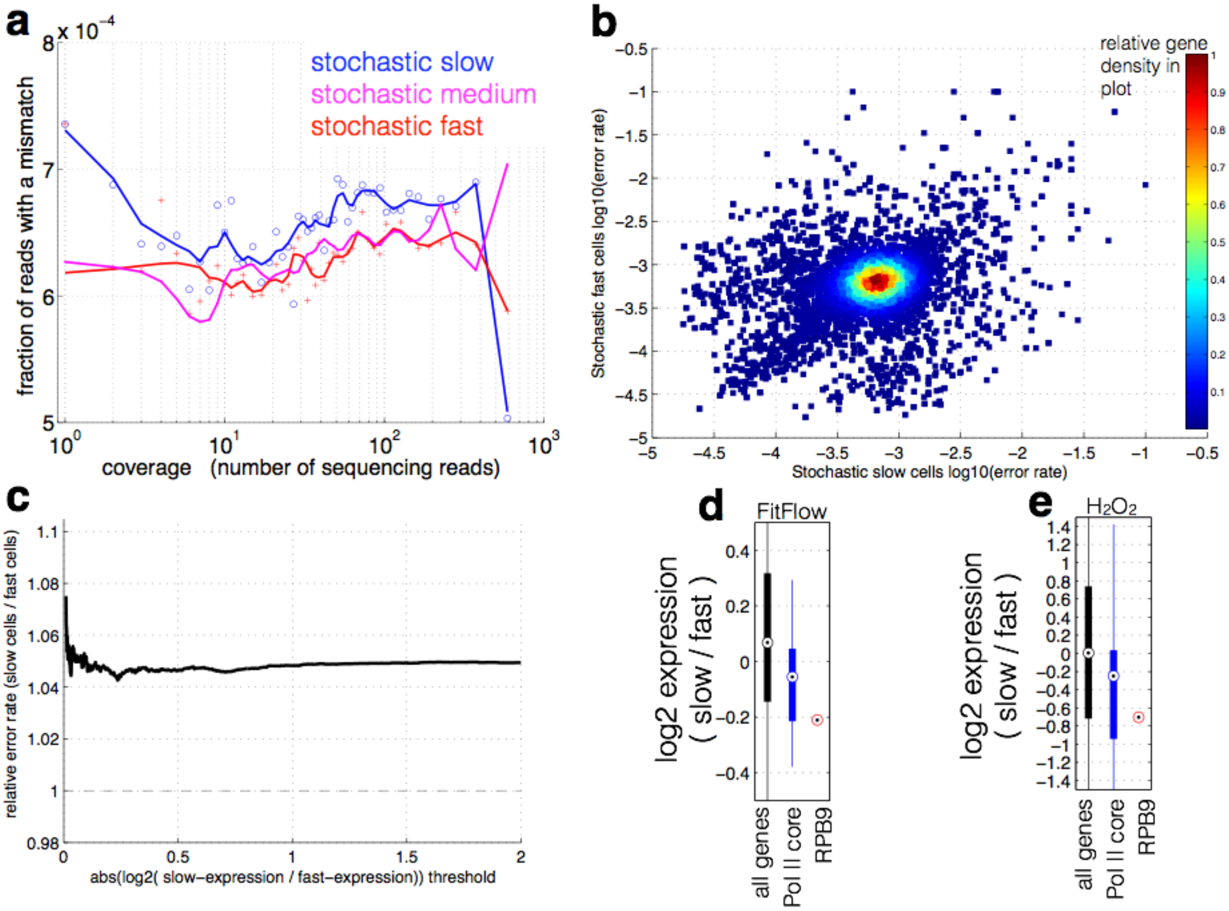

Supplementary Figure 17. Slow growing subpopulations have more RNA polymerase errors independent of the level of transcription. (a) RNA-seq reads with stochastically slow, medium and fast were aligned to the genome and the number of reads and mismatches at each position were counted. In order to test for biases in error rate as a function of transcription level, location in the genome were binned by expression level, and the error rate at each expression level was determined by summing the total number of reads and the total number of errors at all genomic regions with the same expression level. Slow growing subpopulation have more mismatches at all levels of RNA expression (paired t-test, $p=7e^{-8}$), while stochastically fast and medium growing cells have the same error rate (paired t-test, p=0.9). There is no significant difference between the error rates in slow and fast as a function of transcription level. (**b**) There is a small correlation between error rates in stochastically slow and fast cells (R=0.3). We were unable to identify any GO terms, expression level, or any other features associated with differential error rates between growth rates. (**c**) The RNA error rate increase is not due to changes in what is being expressed. In order to see if the differential error rate between stochastically slow and fast cells is due to differential gene expression, errors were only considered if coming from genes with similar expression levels between slow and fast cells. A varying cutoff (x-axis) was chosen, so that only genes with differential expression <= log2(x-axis) were used in the analysis. The ratio between error rates in slow and fast cells (y-axis) is not dependent on differential gene expression (equal error rates would be a line at y=1). **(d,e)** RPB9 is more down-regulated in slow growing cells than the rest of the RNA Pol II complex. Shown are all genes, all members of the RNA Polymerse II complex in yeast, and the fold-change for both DST1 and RBP9.

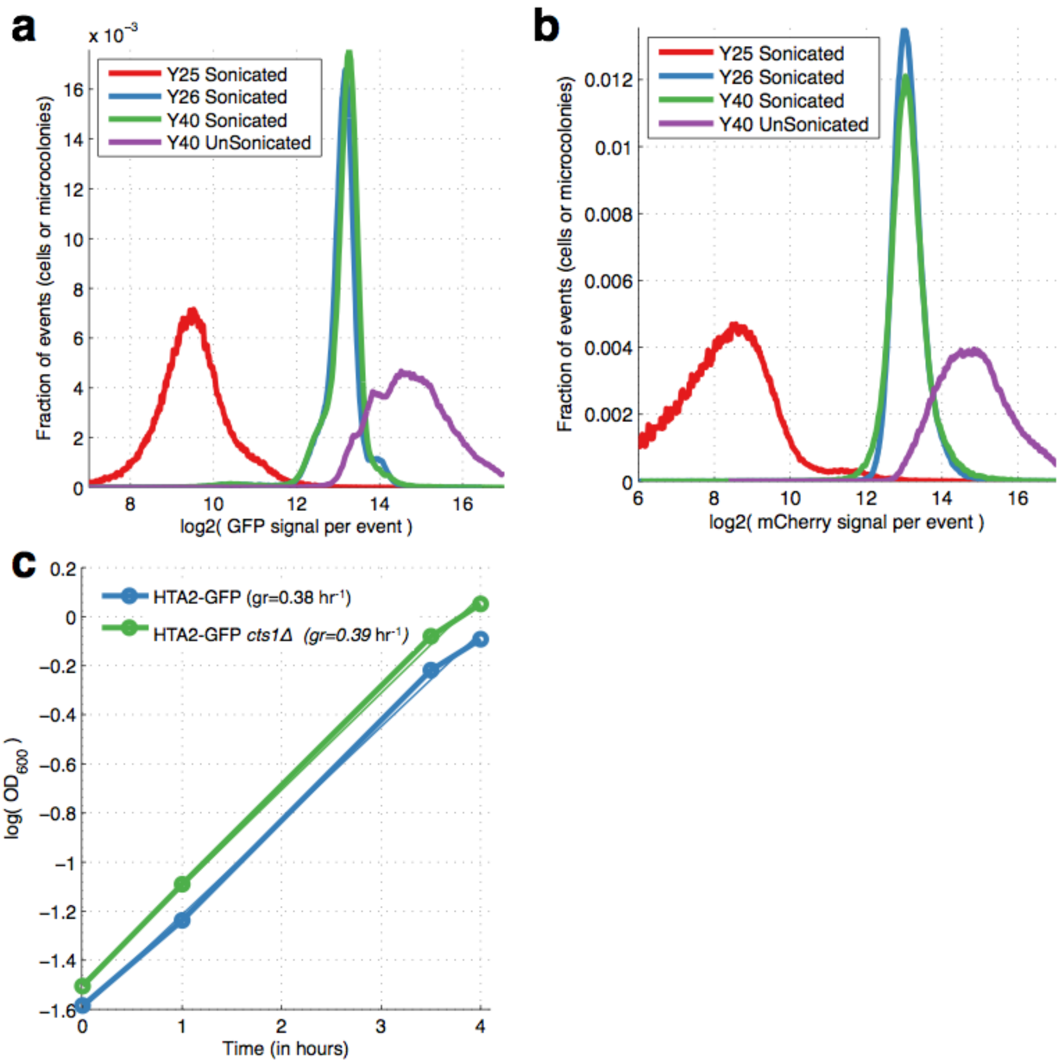

Supplementary Figure 18. *cts1* cells do not have a growth defect or a change in fluorescence distribution. (a,b) A HTA2-GFP TEFpr-mCherry strain (Y26) and an isogenic strain that is also cts1::KanMX (Y40) were grown in SCD and the GFP and mCherry distributions were measured with and without sonication. Strain Y25 expresses neither GFP nor mCherry and is shown as a negative control. Both GFP and mCherry distributions for sonicated Y26 ($CTS1^{+}$) and Y40 (*cts1*) are identical. **(c)** Strains Y26 (blue) and Y40 (green) have the same growth rate when grown in 6ml SCD at 30C.

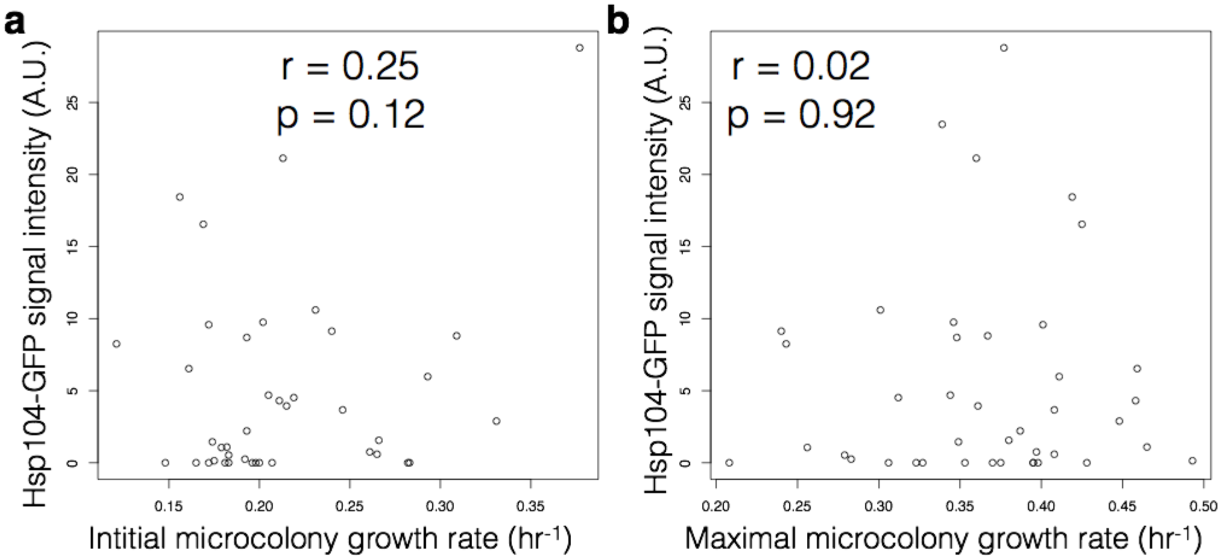

Supplementary Figure 19. **Hsp104-GFP signal does not correlate with microcolony growth rate.** Hsp104-GFP cells were grown to mid-log phase and imaged in brightfield and GFP every 45 minutes using time-lapse microscopy. GFP signal intensity does not correlate with growth rate during the first 90 minutes (a) or maximum microcolony growth rate (b).

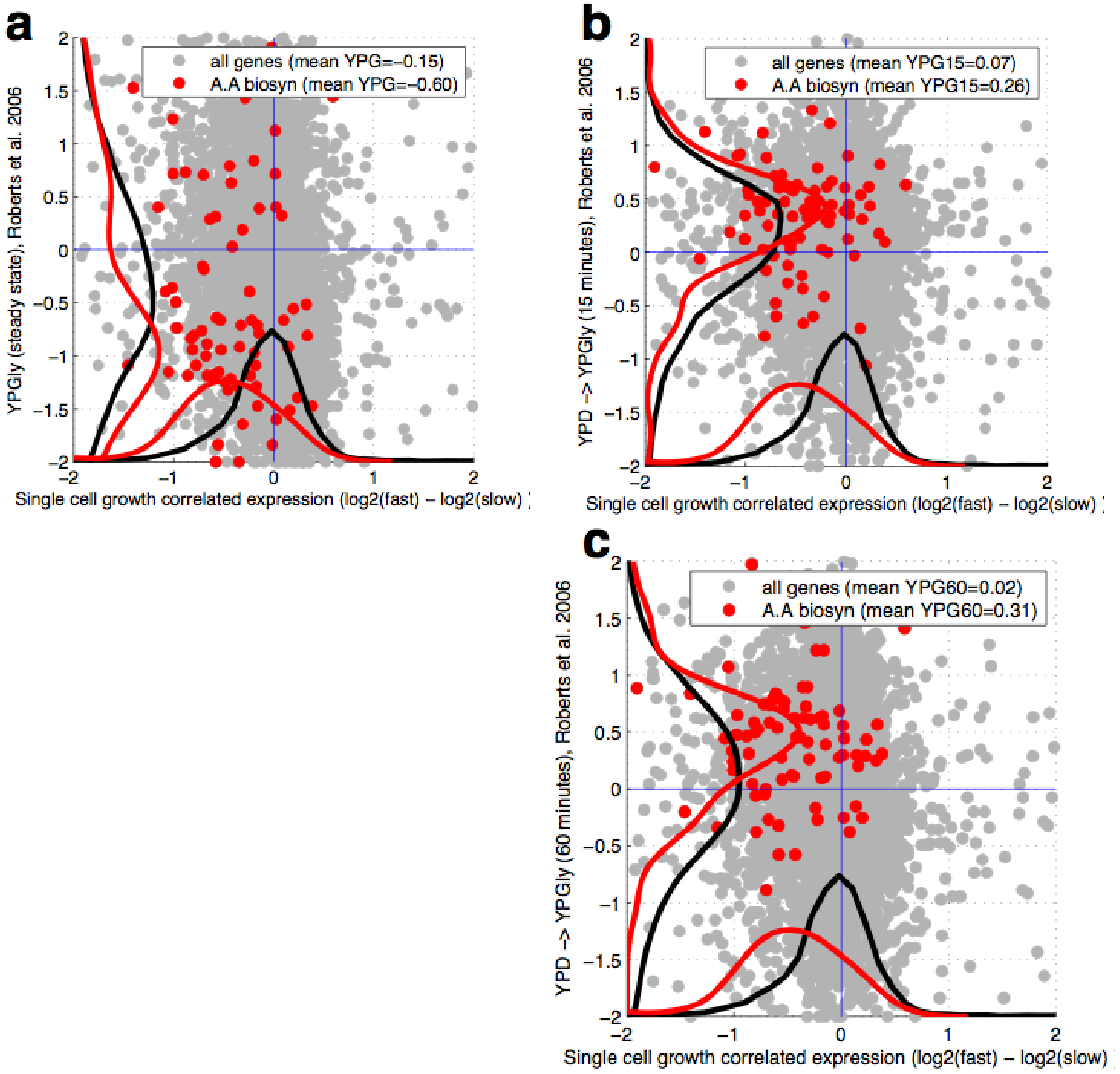

Supplementary Figure 20. **Amino acid biosynthesis genes are up-regulated immediately upon the switch from glucose to glycerol, but not at steady-state growth on glycerol. (a)** A comparison of FitFlow data (x-axis) and steady-state growth on glycerol shows that AA biosynthesis genes are more highly expressed in the slow growing subpopulation but have lower expression during continuous growth in glycerol, similar to the slow growth in Brauer et al. **(b,c)** During the first 60 minutes following the shift from glucose to glycerol, AA biosynthesis genes are up-regulated, similar to the slow growing subpopulation.
